# Agr Quorum Sensing influences the Wood-Ljungdahl pathway in *Clostridium autoethanogenum*

**DOI:** 10.1038/s41598-021-03999-x

**Published:** 2022-01-10

**Authors:** Pawel Piatek, Christopher Humphreys, Mahendra P. Raut, Phillip C. Wright, Sean Simpson, Michael Köpke, Nigel P. Minton, Klaus Winzer

**Affiliations:** 1 Present Address: Department of Biotechnology and Nanomedicine, SINTEF Industry, 7465 Trondheim, Norway; 2 BBSRC/EPSRC Synthetic Biology Research Centre (SBRC), School of Life Sciences, University Park, The University of Nottingham, Nottingham, UK; 3 Department of Chemical and Biological Engineering, The ChELSI Institute, University of Sheffield, Mappin Street, Sheffield, S1 3JD UK; 4 University of Southampton, University Road, Southampton, SO17 1BJ UK; 5 LanzaTech Inc., 8045 Lamon Ave, Suite 400, Skokie, IL 60077 USA

**Keywords:** Microbial communities, Molecular engineering, Proteomics, Metabolic engineering

## Abstract

Acetogenic bacteria are capable of fermenting CO_2_ and carbon monoxide containing waste-gases into a range of platform chemicals and fuels. Despite major advances in genetic engineering and improving these biocatalysts, several important physiological functions remain elusive. Among these is quorum sensing, a bacterial communication mechanism known to coordinate gene expression in response to cell population density. Two putative *agr* systems have been identified in the genome of *Clostridium autoethanogenum* suggesting bacterial communication via autoinducing signal molecules. Signal molecule-encoding *agrD1* and *agrD2* genes were targeted for in-frame deletion. During heterotrophic growth on fructose as a carbon and energy source, single deletions of either gene did not produce an observable phenotype. However, when both genes were simultaneously inactivated, final product concentrations in the double mutant shifted to a 1.5:1 ratio of ethanol:acetate, compared to a 0.2:1 ratio observed in the wild type control, making ethanol the dominant fermentation product. Moreover, CO_2_ re-assimilation was also notably reduced in both hetero- and autotrophic growth conditions. These findings were supported through comparative proteomics, which showed lower expression of carbon monoxide dehydrogenase, formate dehydrogenase A and hydrogenases in the ∆*agrD1*∆*agrD2* double mutant, but higher levels of putative alcohol and aldehyde dehydrogenases and bacterial micro-compartment proteins. These findings suggest that Agr quorum sensing, and by inference, cell density play a role in carbon resource management and use of the Wood-Ljungdahl pathway as an electron sink.

## Introduction

Bacteria have evolved a diverse array of signalling mechanisms that aid in environmental adaptation^1–4^. Some of these systems allow cells to “sense” each other, and coordinate gene regulation in a concerted response with respect to cell population density^5^. This phenomenon is commonly referred to as quorum sensing (QS), with research mainly focusing on bacteria with the first occurrences observed in marine species and later, throughout Gram-positive and negative species^6,7^. QS systems play a significant role in triggering physiological actions that include biofilm formation, motility, bioluminescence, sporulation and production of virulence factors^1,2^. However, few studies have reported and described specific QS systems in biotechnologically relevant species, particularly in *Clostridium* species, known for their solventogenic properties^8–11^. In the historically important *Clostridium acetobutylicum*, two principal QS systems are present, the RRNPP and accessory gene regulator (Agr) systems^10–12^. Both systems serve important roles in sporulation, granulose formation and solventogenesis, all of which serve a central function in the organism’s survival strategies^10,11^. A recent study examining the RRNPP system in *Clostridium saccharoperbutylacetonicum* similarly described its role in solventogenesis, motility, and sporulation^9^. Besides the Agr and RRNPP system, a class of auto-inducing, post-translationally modified peptides were identified and characterised in the solventogenic *Clostridium beijerinckii* and the gas fermenting *Clostridium ljungdahlii* and were both reported to be involved in sporulation and solventogenesis^8^. Next-generation sequencing has allowed us to identify homologous QS genes of various systems conserved across many *Clostridium* species, including industrially important acetogens, which are considered to be promising biocatalysts for sustainable chemical and fuel production^13–16^.

In recent years, there has been a considerable drive towards the development of sustainable platform chemicals through microbial synthesis gas (syngas) fermentation. This novel carbon-capture strategy employs acetogenic bacteria, which are naturally capable of fermenting syngas components into a range of valuable fermentation products^17,18^. Acetogens are typified for their unique metabolism that enables CO, H_2_ and CO_2_ autotrophy, which are the primary components of industrial syngas^19–21^. *Clostridium autoethanogenum* is a Gram-positive, chemolithotrophic, motile anaerobe, and considered a model acetogen with proven industrial utility. When cultured autotrophically on syngas, its primary native products include acetate, ethanol, 2,3-butandiol and lactate^22,23^. The ancient Wood-Ljungdahl pathway (WLP) is considered the primary mechanism that drives autotrophic formation of fermentative products and biomass. In brief, the WLP comprises of separate methyl and carbonyl branches that converge to produce a single molecule of acetyl-CoA, whereby the methyl branch delivers a methyl group and the carbonyl branch delivers an enzyme-bound CO molecule which combine with coenzyme A to form acetyl-CoA via acetyl-CoA synthase (ACS). H_2_ provides the required reducing equivalents during lithoautotrophic growth on CO_2_, whereas during CO-based growth, reducing equivalents are derived via CO oxidation through carbon monoxide dehydrogenase (CODH)^23,24^.

Interestingly, the WLP does not generate net ATP through substrate-level phosphorylation (SLP), but instead relies on chemiosmotic mechanisms such as the membrane-bound, ferredoxin-NAD:oxidoreductase complex (Rnf) that uses reduced ferredoxin-dependent proton translocation to drive the ATP synthase complex into generating ATP^25–27^. Under heterotrophic conditions, *C. autoethanogenum* converts sugars and other organic compounds into biomass and fermentative end products, predominantly acetate and ethanol, which contribute towards ATP generation via SLP^28–30^. Glycolytic breakdown of sugars also generates reducing equivalents and CO_2_ which then feed into the WLP, thereby re-oxidising electron carriers and conserving additional energy^28^.

Studies examining *C. autoethanogenum’s* metabolism have greatly benefitted from the usage of available sequenced genomes and collective “-omic” technologies^31–36^, and comprehensive genetic tools and metabolic models have allowed for efficient genome editing with respect to stress tolerance, improved fermentation yields and increasing the repertoire of various product outputs^37–41^. Emphasis on understanding the WLP has elucidated much in the way of energy conservation, redox metabolism and carbon utilisation^42–45^, which has laid out incentives for furthering metabolic efficiency and industrial utility. Yet despite these advances, other significant physiological functions are still not yet fully understood such as cell–cell communication systems. *C. autoethanogenum* harbours both RRNPP-like and Agr QS genes within its genome. Examination of supplementary transcriptomic data provided by Marcellin *et. al.* reveals that *agr* genes exhibit relatively high RNA counts during auto- and heterotrophic growth conditions^34^, even though their function remains unclear. Similar observations have been made in *C. autoethanogenum*’s close relative, *C. ljungdahlii*, whose transcriptome during fructose and syngas-based fermentation revealed upregulation of both QS and sporulation associated genes^46^.

The Agr QS system was first described in *Staphylococcus aureus* and is arranged in five distinct elements represented through genes, *agrA, agrB, agrC, agrD* and RNAIII^47^. The translated functions of these genes can be described as follows: AgrD represents an autoinducing peptide (AIP) precursor which, following AgrB-mediated processing and extra-cellular release, in its final, mature form conforms to a cyclic structure. Together, AgrA and AgrC comprise a two-component system mediating AIP receptor/response functions via phosphorelay. The AgrC receptor is a membrane-bound histidine kinase which phosphorylates AgrA in response to AIP binding once a certain AIP threshold concentration has been reached. Phosphorylated AgrA binds to two promoters, P2 and P3, activating the divergently transcribed *agrBDCA* operon and the regulatory RNAIII. This action results in a positive feedback loop to produce more AIPs and subsequent expression of RNAIII, the latter then activating and inhibiting target gene expression^47,48^. RNAIII appears to be a typical feature of staphylococcal Agr systems and has not been described for the few clostridial systems studied so far.

The Agr system is not as well characterised in *Clostridium* spp., but evidence suggests it is widely conserved^49^, and several pathogenic species utilise full, multiple, or partial arrangements of Agr-like systems similarly to *S. aureus*, with examples observed in *Clostridium perfringens*, *Clostridium botulinum, Clostridium difficile*^50–53^ and several more. In many cases, these pathogens have specific uses for their respective Agr systems, but these systems are understood to play a role in sporulation, toxin production, and biofilm formation^50,51,54^. Despite this, not much is known about the Agr system in other clostridial species.

Several acetogenic clostridia contain genes annotated to encode putative Agr proteins. However, to date none of these putative Agr systems have been experimentally investigated. The aim of this study was to examine the role of both Agr QS systems in *C. autoethanogenum* and to establish whether they play a part in regulating the organism’s physiology and metabolism. This was achieved through the deletion of both *agrD1* and *agrD2* genes, encoding putative AIPs, with the intention to disable Agr QS function.

## Results

### *C. autoethanogenum* possesses two putative *Agr* systems

**Figure 1:**
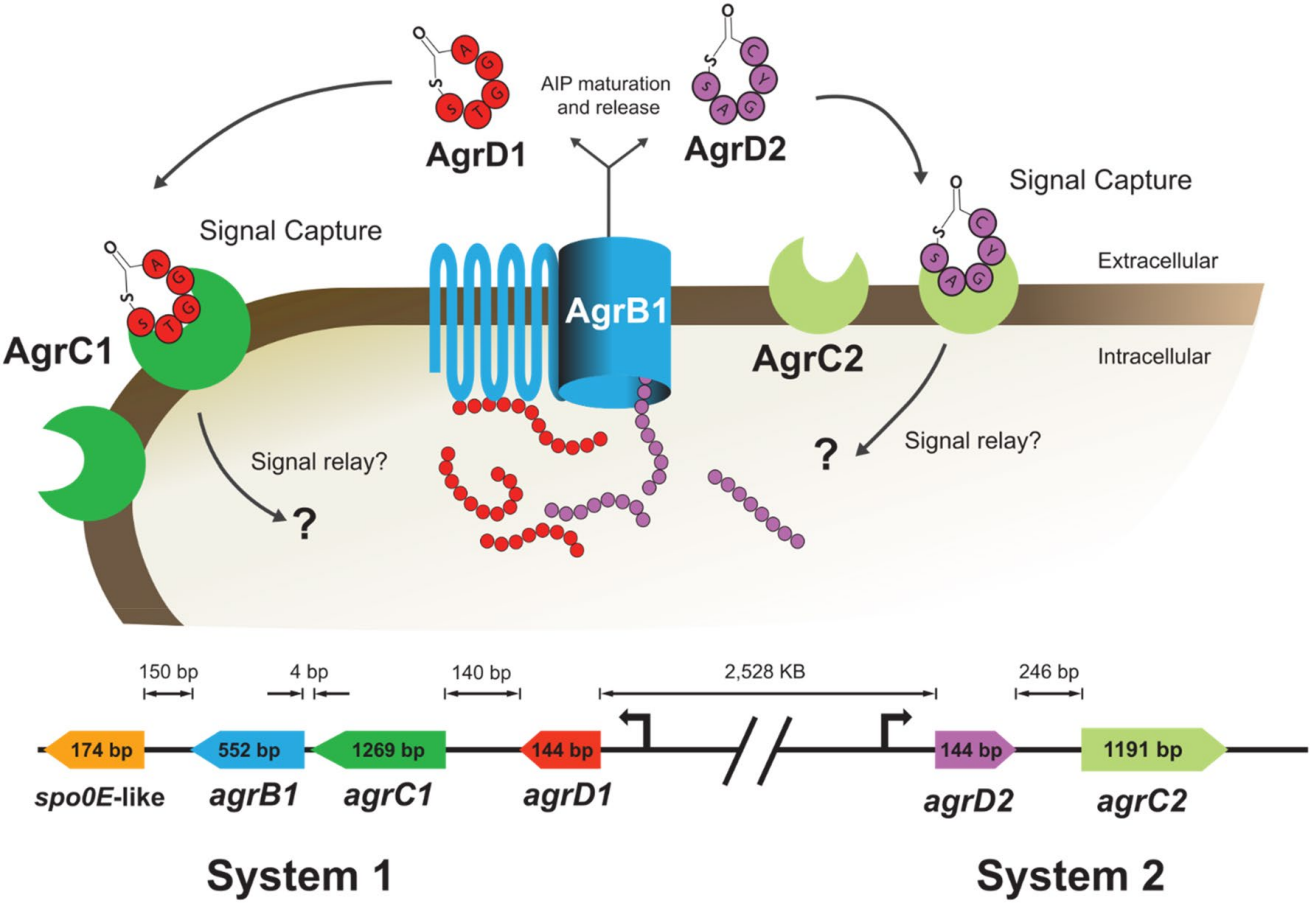
Schematic of the proposed Agr QS systems in *C. autoethanogenum*. Two separate, putative *agr* operons are present in the genome. Both are incomplete: operon 1 is missing the response regulator encoding gene *agrA*, whereas operon 2 is lacking both *agrB1* and *agrA* homologs. The precursors of two distinct autoinducing peptides (AIPs) are encoded by *agrD1* and *agrD2*, respectively. Export and processing of both precursors is hypothesised to be mediated by membrane associated AgrB1. AgrC1 and AgrC2 are putative histidine kinases which act as receptors for AgrD1 and AgrD2 derived AIPs, respectively. In other Agr systems, their role is to phosphorylate their cognate AgrA response regulators. In *C. autoethanogenum*, these may be encoded elsewhere on the chromosome. A *spo0E*-like gene (CLAU_0813) is located downstream of *agrB1*, this arrangement is also observed in several other acetogenic clostridia.

Two putative *agr* operons have been annotated in *C. autoethanogenum*, referred to here as Agr systems 1 and 2 (Fig. 1). Both systems appear incomplete in contrast to the previously described Agr system in *S. aureus*^47^, or that of the solvent producing *C. acetobutylicum*^11^. System 1 consists of three genes, *agrD1*, *agrC1* and *agrB1* (CLAU_0816, CLAU_0815, and CLAU_0814, respectively), but lacks an *agrA* response regulator gene. Located immediately downstream of the proposed operon, and potentially a part of it, is a small *spo0E*-like gene (CLAU_0813). System 2 comprises only two genes, *agrD2* and *agrC2* (CLAU_3094 and CLAU_3095). Since no other *agrB1* homologue could be identified in the genome, it seems that processing of both AgrD1 and AgrD2 signalling peptides relies on the System 1 encoded AgrB1 (Fig. 1).

A bioinformatics survey revealed that operons homologous to *C. autoethanogenum*’s System 1 and System 2 are also present in several other clostridial acetogens. System 1 operons share high similarity in the encoded protein sequences and are found in the closely related *Clostridium ljungdahlii*, *Clostridium coskatii*, *Clostridium ragsdahlii*, *Clostridium drakei* and *Clostridium scatologenes* (Supplementary information, Table S4). In the former three, a *spo0E*-like gene is also present. The aforementioned species also contain the *agrD2C2* operon, although the surrounding genomic context deviates for *C. coskatii*, *C. ragsdahlii* and *C. scatologenes*. The *C. autoethanogenum*’s AgrD1 and AgrD2 AIPs share considerable similarity in protein sequences amongst the above examples, especially in the conserved Pro-X-X-Pro motif region that has been suggested to represent the recognition site in which AgrD AIPs interacts with AgrB1^55^. However, each species AgrD AIP has a unique 5-membered lactone ring structure which is thought to bind to respective AgrC receptors^48,56^ (Supplementary information, Table S5).

### Generation and complementation of *agrD* mutants

To study the role of the Agr system in *C. autoethanogenum*, both *agrD* signalling genes were targeted for in-frame deletion using allelic-exchange techniques to abolish putative signalling capabilities^57–59^. Genes *agrD1* (CLAU_0814) and *agrD2* (CLAU_3176) in each *agr* operon were either targeted individually or in tandem. In-frame deletion methods removed approximately 80% of each *agrD* gene, leaving a 30-bp non-functional sequence. The starting point for this strategy was a *pyrE* defective *C. autoethanogenum* strain, which due to this defect displayed uracil auxotrophy and 5-fluorouracil resistance^60^. The reason for using this strain lay in the ease with which chromosomal complementation of the introduced *agrD* deletions could be achieved: repair of the *pyrE* defect in the final step of the procedure restored uracil prototrophy and could be done either on its own or coupled to co-integration of an intact copy of the previously targeted *agrD* gene immediately downstream of *pyrE*. Hence, the final strains carried the described *agrD* modifications, but were otherwise identical to the wild type (WT). Three *agrD* mutant permutations were created in this manner consisting of two single knockouts, and a double knockout respectively: Δ*agrD1*, Δ*agrD2* and Δ*agrD1D2*. Two further Δ*agrD1D2* independent mutants were created later in the study, and designated Δ*agrD1D2_1* and Δ*agrD1D2_2* and were compared against the original double knockout mutant and WT. As stated above, each mutant was *pyrE* corrected to regain uracil autotrophy before full phenotypic characterisation^57,61^ (Supplementary information, Fig. S1).

Chromosomal complementation was performed using *agrD* double mutant, Δ*agrD1D2_*Δ*pyrE,* which was conjugated with *pyrE* repair plasmids, pMTLCH20-D1 and pMTLCH20-D2 that contained *agrD1* and *agrD2* genes, respectively. Either *agrD* gene was chromosomally integrated downstream of the simultaneously repaired *pyrE* gene and was expressed via the *pyrE* operon promoter instead of its native *agr* promoter. Successful construction of mutants and complemented mutants was routinely verified by PCR and Sanger-sequencing, and further confirmed through whole genome sequencing as described in the methods. Complemented mutants were designated Δ*agrD1D2* + *D1comp* and Δ*agrD1D2* + *D2comp* respectively and phenotypically characterised.

### Phenotypic characterisation of *agrD* mutants in heterotrophic conditions

**Figure 2:**
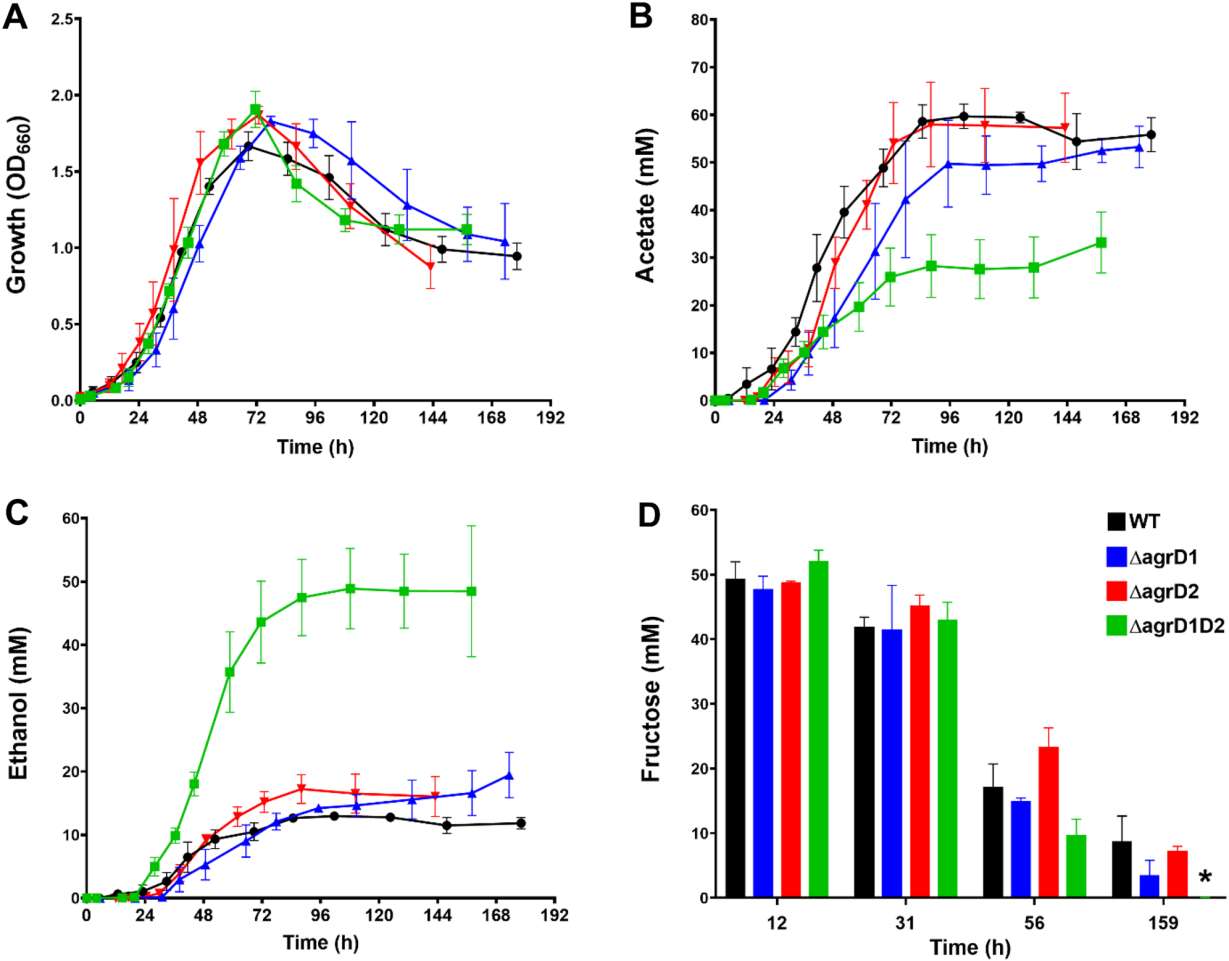
Growth on PETC with 50 mM fructose showing metabolic product profiles comparing single and double knockout *agrD* mutants with respect to WT showing; (**A**) Growth profile, (**B**) acetate production, (**C**) ethanol production and (**D**) fructose consumption. Black circles, WT (n = 5); Blue triangles, Δ*agrD1* (n = 3); Red inverted triangles, Δ*agrD2* (n = 4); Green squares, Δ*agrD1D2* (n = 6). Colour representations corresponds similarly to bar graph (**D**). Error bars indicate standard error of the mean, * equates to a ≤ 0.05 P value of statistical difference to the WT.

Heterotrophic growth and fermentation profile assessments of the Δ*agrD1*, Δ*agrD*2 and Δ*agrD1D2* mutants against the WT were performed for cultures grown in PETC medium containing 50 mM d-fructose at 37 °C for approx. 175 h. Transition from exponential to stationary phase was typically seen after 48 h and peak optical densities were similar for all strains (Fig. 2A).

Determining fermentation product profiles focused on ethanol and acetate titres (Fig. 2B,C), whereas fructose consumption was determined at specific time points of 12, 31, 56 and 159 h (Fig. 2D). Overall, acetate production profiles were similar for single knockout mutants Δ*agrD1* and Δ*agrD2* and the WT, and although titres appeared slightly lower for Δ*agrD1* there were no statistical differences against the WT, with final concentrations of acetate comprising of 53.3 ± 4.4 mM (P = 0.7), 57.3 ± 7.3 mM (P = 0.9), and 55.8 ± 3.6 mM, respectively (Fig. 2B). Similarly, no major differences were observed in final ethanol concentrations of Δ*agrD1* and Δ*agrD2* and the WT that reached 19.5 ± 3.6 mM (P = 0.17), 16.1 ± 3.1 mM (P = 0.42) and 11.8 ± 0.9 mM, respectively (Fig. 2C). However, notable differences were observed for the Δ*agrD1D2* mutant in comparison to the WT. Ethanol concentrations increased fourfold to a final concentration of 48.9 ± 10.3 mM, whereas the final acetate concentration was around 1.7-fold lower, reaching only 33.2 ± 6.4 mM. Fructose consumption was measured using HPLC at specified time points and was noted to be similar amongst all strains, although the Δ*agrD1D2* had consistently consumed all fructose at the end of the experiment, whereas concentrations of 3.6 ± 2.3 mM, 7.3 ± 0.7 mM, and 8.7 ± 3.9 mM remained for Δ*agrD1*, Δ*agrD2* and WT, respectively, Fig. 2D. Other fermentative products such as lactate and 2,3-butanediol were only produced in very small amounts, (final concentrations ranging from 4 to 6 mM respectively) and showed no significant differences amongst strains (not shown).

**Figure 3:**
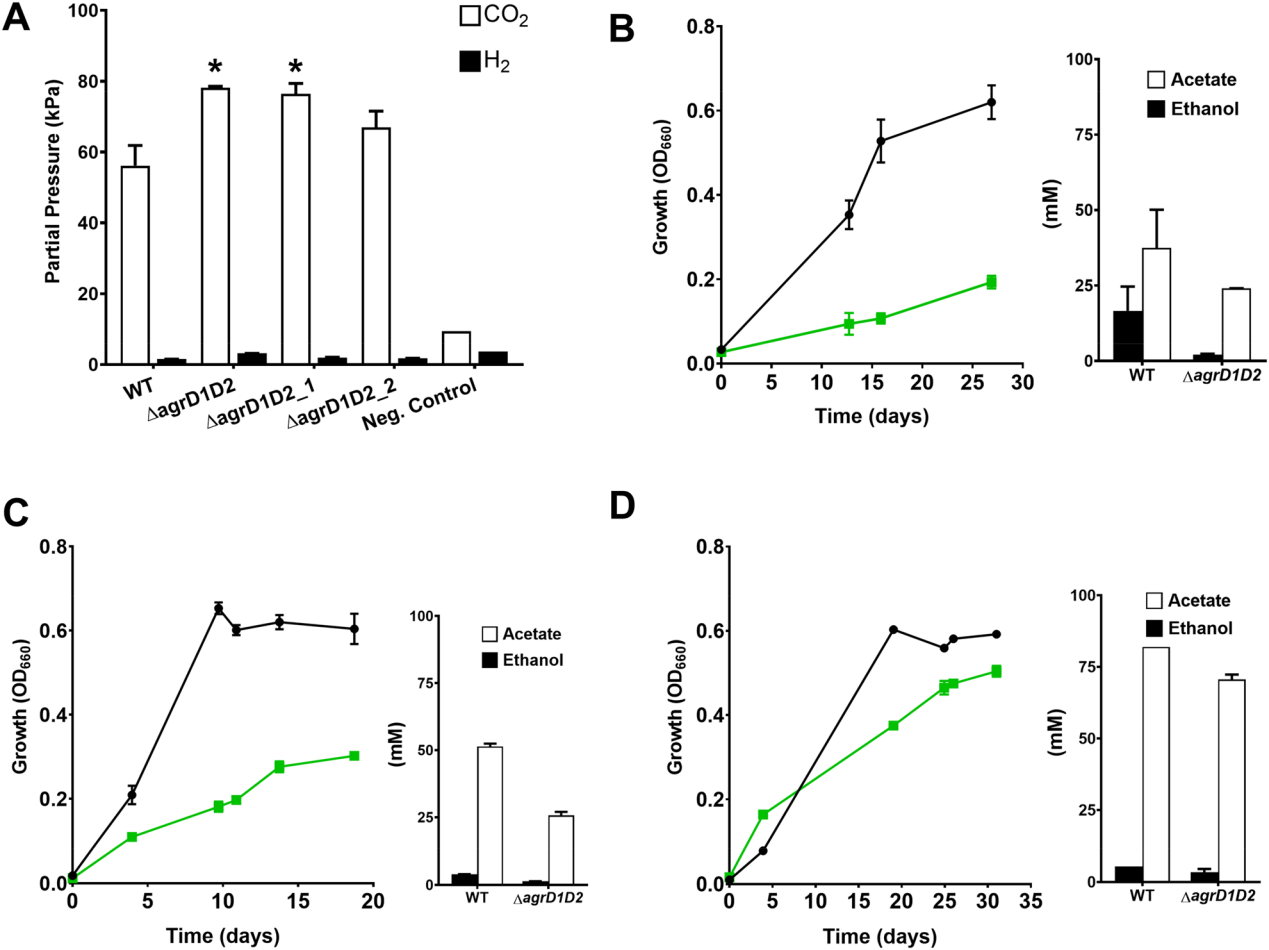
Diminished CO_2_ re-assimilation and autotrophic behaviour of Δ*agrD1D2* mutants represented through; (**A**) Comparison of CO_2_ partial pressures of fructose-grown cultures after 200 h in three independent Δ*agrD1D2* mutants against the WT (n = 4), Δ*agrD1D2* (n = 3), Δ*agrD1D2_1* (n = 4), Δ*agrD1D2_2* (n = 4) and negative control (n = 1); the negative control represented WT cultures without fructose addition. (**B**–**D**) Adaptive growth on 200 kPa CO with fermentation product profiles of WT (Black circles) and Δ*agrD1D2* (Green squares) that represent first (**A**), second (**B**), and third (**C**) subcultures of WT (n = 2) and Δ*agrD1D2* (n = 2) cultures, growing over a period of 29, 19 and 31 days respectively. Main metabolic products, acetate and ethanol were measured at the end of each fermentation. All error bars indicate standard error of the mean, P values equate to: * ≤ 0.05 of statistical difference to the WT.

CO_2_ is liberated as a by-product during fructose-based fermentation and is partially reassimilated for WLP-dependent acetate formation^42^. Serum flasks were routinely monitored for total pressure with a handheld pressure gauge. Visual inspection and sampling of flasks had suggested that Δ*agrD1D2* flasks in contrast to single mutants and WT, had built up higher total gas pressures towards the end of fermentation experiments. When pressure was checked in one particular experiment, total pressures of 159, 114 and 115 kPa were found for Δ*agrD1D2*, Δ*agrD2* and WT respectively (the Δ*agrD1* mutant was not analysed during this particular experiment). A GC-based analysis of the serum flask head space was performed, and CO_2_ observed at significantly higher partials pressures in Δ*agrD1D2* flasks which reached 99.0 ± 2.1 kPa, compared to the WT and the Δ*agrD2* mutant which only reached 33.4 ± 1 kPa and, 36.3 ± 1 kPa, respectively. To provide further evidence that these results were due to the complete inactivation of Agr QS, two further double knockout mutants (Δ*agrD1D2_1* and Δ*agrD1D2_*2) were created. The original Δ*agrD1D2* mutant was obtained by introducing the Δ*agrD2* mutation into mutants with a Δ*agrD1* background, whereas the Δ*agrD1D2_1* and Δ*agrD1D2_2* mutants were generated by inactivating *agrD1* in the Δ*agrD2* background. The headspace composition produced by each mutant was measured after 200 h and an increase of CO_2_ partial pressure was again observed for the original Δ*agrD1D2* double mutant, as well as the two newly generated Δ*agrD1D2_1* and Δ*agrD1D2_2*, with 78.2 ± 0.4 kPa (P = 0.02), 76.4 ± 3 kPa (P = 0.02) and 70.0 ± 4.6 kPa (P = 0.19), respectively compared to 56.2 ± 5.7 kPa for the WT (Fig. 3A). Traces of H_2_ were also measured (Fig. 3A), serum flasks contained a starting H_2_ pressure of 3.7 kPa, but the resolution in differences in consumed H_2_ amongst all strains were too minor to be considered significant. At the end of fermentation, the Δ*agrD1D2,* Δ*agrD1D2_1* and Δ*agrD1D2_2* exhibited H_2_ amounts of 3.2 ± 0.1 kPa, 2 ± 0.2 kPa and 1.8 ± 0.2 kPa respectively against the WT, 1.6 ± 0.1 kPa. The negative control remained at 3.7 kPa.

### Characterisation of autotrophic metabolism

The apparent lack or reduction of CO_2_ assimilation by Δ*agrD1D2* mutant cultures grown on fructose led to a comparison of autotrophic growth between the Δ*agrD1D2* mutant and the WT. Cultures were grown in 250 ml serum flasks containing 50 ml PETC medium and supplied with 200 kPa CO for periods between 19 to 31 days (Fig. 3B–D), inoculated at a starting OD_660_ of 0.01 from washed PETC precultures grown on fructose. The first batch revealed diminished growth of the Δ*agrD1D2* strain compared to the WT, with strains reaching a maximum OD_660_ of 0.19 ± 0.02 and 0.62 ± 0.04, respectively. The two strains also differed in their final product titres and their respective ratios. Whilst the WT had produced about twice as much acetate (37.5 ± 12.7 mM) than ethanol (16.6 ± 8.0 mM), the Δ*agrD1D2* mutant, in contrast to its behaviour on fructose, had generated mostly acetate (24 ± 0.1 mM) and only minor amounts of ethanol (2.1 ± 0.2 mM). This suggested that the mutant was less capable of switching from heterotrophic to fully autotrophic growth. After 27 days, strains were subcultured into a fresh, second batch of serum flasks and grown under the same conditions revealing a slight growth improvement for Δ*agrD1D2* which reached a final OD_660_ of 0.3 ± 0.01 compared to 0.6 ± 0.04 of the WT. Whilst the final product titres had not changed much for the double mutant (25.7 ± 1.3 mM acetate, 1.3 ± 0.1 mM ethanol), the WT showed some adaptation and produced mainly acetate (51.4 ± 1.1 mM) and only minor amounts of ethanol (3.9 mM). The final subculture showed that the Δ*agrD1D2* mutant had adapted further and now performed almost as well as the WT with final densities of OD_660_ of 0.5 and 0.59 ± 0.01, respectively. The final acetate and ethanol concentrations of the third successive batch cultures were also measured, with the Δ*agrD1D2*’s acetate and ethanol titres being 70.6 ± 1.7 mM and 3.4 ± 1.1 mM, similar to that observed for the WT, 82 mM and 5.4 mM, respectively, (Fig. 3B–D).

### Complementation of Δ*agrD1D2* mutants

**Figure 4:**
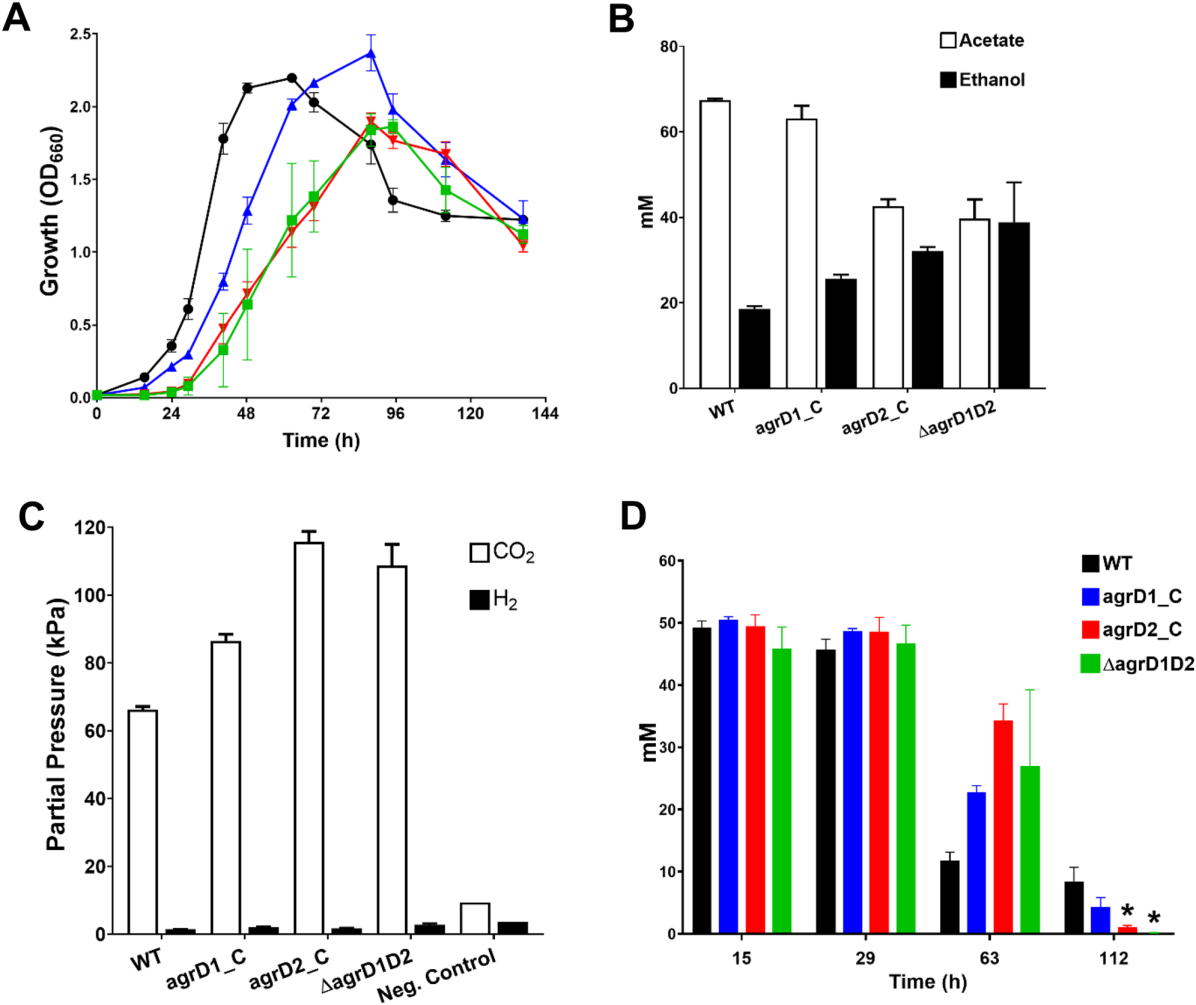
Growth, fermentation, and CO_2_ pressure profiles of complemented *agrD* mutants revealing; (**A**) Growth profile, (**B**) acetate and ethanol production after 136 h, (**C**) CO_2_ and H_2_ pressures measured after 136 h and (**D**) fructose consumption. Black circles, WT (n = 3); Blue triangles, Δ*agrD1D2* + *D1comp* (n = 3); Red inverted triangles, Δ*agrD1D2* + *D2comp* (n = 3); Green squares, Δ*agrD1D2* (n = 3). The negative control represented cultures without fructose addition. All error bars indicate standard error of the mean, P values equate to: * ≤ 0.05 of statistical difference to the WT.

As single *agrD* knockout mutants showed no discernible phenotypic differences compared to the WT, only the Δ*agrD1D2* double knockout mutant was complemented. As detailed above, this was done by inserting intact copies of either *agrD1* or *agrD2* immediately downstream of the *pyrE* gene on the chromosome, thereby generating strains Δ*agrD1D2* + *D1comp* and Δ*agrD1D2* + *D2comp*, respectively. The tested strains were then examined in PETC medium containing 50 mM D-fructose and their fermentation profiles were compared to the WT and Δ*agrD1D2* mutant. As shown in Fig. 4A, complementation with *agrD1* resulted in acetate and ethanol titres similar to those observed for the WT: the final acetate and ethanol concentrations reached 63.1 ± 3 mM and 25.6 ± 0.9 mM, respectively for the Δ*agrD1D2* + *D1comp* strain, compared to 67.4 ± 0.4 mM and 18.5 ± 0.7 mM respectively for the WT. However, titres for the *agrD2* complemented strain, Δ*agrD1D2* + *D2comp* matched closer to the original Δ*agrD1D2* mutant, with 42.6 ± 1.7 mM acetate and 32.1 ± 1 mM ethanol, respectively. Similarly, headspace gas analysis at the end of the fermentation showed that CO_2_ partial pressures for Δ*agrD1D2* + *D1comp* cultures (86.5 ± 2 kPa) were closer to the WT (66.3 ± 1 kPa), whereas those for the Δ*agrD1D2* + *D2comp* strain (115. 7 ± 3 kPa) resembled the pressures observed for the Δ*agrD1D2* mutant, (108. 7 ± 6.3 kPa) (Fig. 4B). Thus, it appears that chromosomal expression of *agrD1* from the *pyrE* locus was able to closely restore WT characteristics in the double mutant, whereas for unknown reasons this was not the case for the *agrD2* complemented strain.

### Comparison of Δ*agrD1D2* and WT proteomes

**Figure 5:**
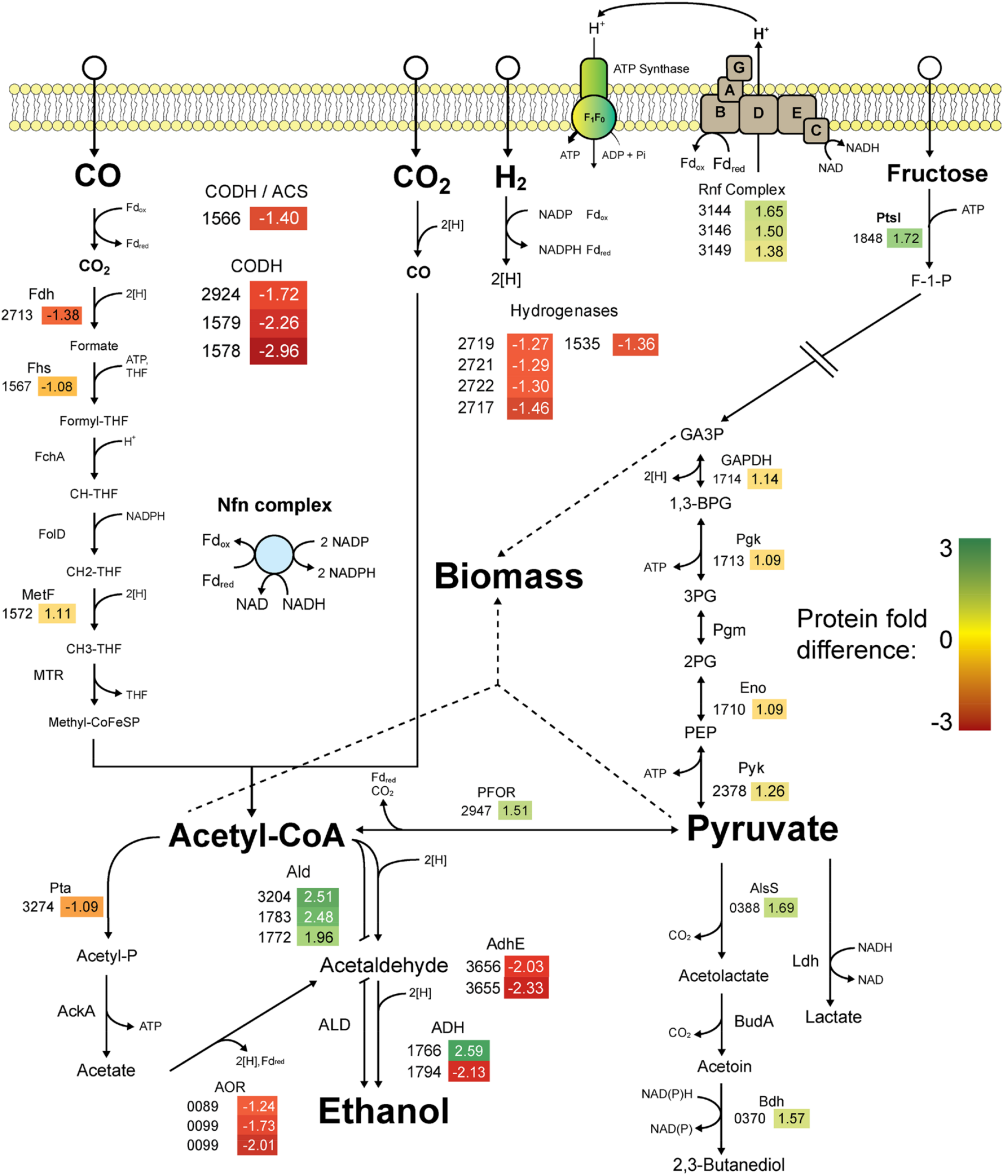
Overview of the relative abundance of the protein expression in Δ*agrD1D2* against WT investigated by quantitative proteomics using iTRAQ. Cultures were grown in PETC medium with 50 mM D-fructose in duplicate to approx. OD_660_ 1.0 before sampling and iTRAQ-based analysis of extracted proteins. The experiment was performed in biological duplicate. Protein/Enzyme abbreviations are given above with gene number IDs on the left, to be preceded with locus tag “CLAU_”. Numerical fold differences on right with colour heatmap indicator: negative values indicate reduced abundance; positive values indicate enhanced abundance of proteins**.** Protein/Enzyme abbreviations: *ACS* acetyl-CoA synthase, *ADH* alcohol dehydrogenase, *AdhE* bifunctional acetaldehyde-CoA/alcohol dehydrogenase, *ALD* acetaldehyde dehydrogenase (acetylating), *AlsS* acetolactate synthase, *AOR* aldehyde:Fd oxidoreductase, *Bdh* 2,3-butanediol dehydrogenase, *BudA* acetolactate decarboxylase, *CODH* carbon monoxide dehydrogenase, *Eno* enolase, *FchA* methenyl-THF cyclohyrolase, *Fdh* formate dehydrogenase, *Fhs* formyl-THF ligase, *FolD* methenyl-THF dehydrogenase, *GAPDH* Glyceraldehyde-3-phosphate dehydrogenase, *Ldh* lactate dehydrogenase, *MetF* methenyl-THF reductase, *MTR* methyltransferase, *PFOR* pyruvate:ferredoxin oxidoreductase, *Pgk* phosphoglycerate kinase, *Pgm* phosphoglycerate mutase, *Pta* phosphotransacetylase, *Ptsl* phosphotransferase system, *Pyk* pyruvate kinase. Hydrogenases, CLAU_2717, 2719, 2721, 2722 represent HytC, HytD, HytA, HytE2 subunits of the HytABCDE1E2 cluster respectively, hydrogenase CLAU_1535 is Hyd Fe-only. Not shown; full fructolytic pathway, full hydrogenase pathways and BMC fold changes.

To better understand the metabolic shift to increased ethanol formation for fructose-grown cells, the Δ*agrD1D2* mutant’s proteome was compared to that of the WT by LC–MS/MS-based iTRAQ quantitation. Cultures were grown on 50 mM fructose in PETC medium and cells were harvested when reaching an OD_660_ of approx. 1.0, as cultures established their typical fermentation patterns (compare Fig. 2). Extracted peptides from the biological duplicates of WT and Δ*agrD1D2* cells were labelled using iTRAQ 4-plex and analysed with MaxQuant software (see Supplementary information). From the 4-Plex iTRAQ experiment, 843 proteins were identified with quantification information with 2 unique peptides at 1% false discovery rate (FDR). Of these 843 proteins, 282 proteins were determined to be statistically differentially regulated (P < 0.0001), with Posterior Error Probability (PEP) of < 0.05 relating to peptide expression levels. Within these 282 quantified proteins (full list in Supplementary information, Sect. 3), 39 proteins with differential abundances relating to *C. autoethanogenum’*s metabolism were categorised into WLP, pyruvate metabolism, glycolysis, energy conservation and fermentation end-product groups (Fig. 5).

Enzymes central to both branches of the WLP, the CODH/ACS with subunit complexes (CLAU_1578 and 1579) and CODH/ACS complexes (CLAU_2924 and 1566), showed reduced abundance for the Δ*agrD1D2* mutant. Several hydrogenase subunits (CLAU_2717, 2719, 2721 and 2722) representing HytC, HytD, HytA, HytE2 subunits respectively, encoded by the *hytCBDE1AE2* cluster, were also observed to be in low abundance alongside a subunit of the [FeFe]-hydrogenase (CLAU_1535). Furthermore, formate dehydrogenase A (CLAU_2713; FdhA), known to form a complex with HytABCDE1E2 (and encoded as part of the *hyt* cluster), was also found in low abundance in the mutant. To independently confirm some of these changes, the overall specific activities for CODH and hydrogenase were determined using crude cell lysates derived from fructose-grown cultures of Δ*agrD1D2* and WT as described in the Methods section. Assays involving hydrogenases and CODH used methyl viologen (MV) as an electron acceptor. WT hydrogenases showed eightfold higher specific activity than Δ*agrD1D2* with 28.6 ± 2 U/mg to 3.5 ± 0.7 U/mg, respectively (P = 0.00006). Similarly, CODH specific activity was twofold higher in the WT than Δa*grD1D2*, with 1.32 ± 0.1 U/mg to 3.5 ± 0.7 U/mg (P = 0.003).

Enzymes involved in alcohol metabolism were divided into two distinct groups: reduced and enhanced abundances for the Δ*agrD1D2* mutant. Bifunctional acetaldehyde-CoA/alcohol dehydrogenases AdhE1 and AdhE2 (CLAU_3655 and 3656), alcohol dehydrogenase (CLAU_1794) and aldehyde ferredoxin oxidoreductases (AOR) (CLAU_0099 and 0089) all were found in low abundance. The latter three enzymes are noted to play a role during autotrophic metabolism^31,34^. Enhanced abundance of enzymes involved in alcohol formation included alcohol dehydrogenases (CLAU_1766 and 3861) and three, mono-functional, acetaldehyde dehydrogenases (CoA acetylating) (CLAU_3204, 1783 and 1772)^59^ (Fig. 5), the genes of which are all linked to clusters encoding putative bacterial microcompartments (BMC). Given that some of the enzymes predicted to contribute to ethanol production were increased in the Δ*agrD1D2* mutant whereas others were reduced, overall NADH-dependent alcohol dehydrogenase activities were determined. Alcohol dehydrogenase specific activity was measured using acetaldehyde as a substrate, with NADH as an oxidative cofactor, and showed threefold higher activity in Δ*agrD1D2* in comparison to the WT, with 0.015 ± 0.002 U/mg (P = 0.002) and 0.005 ± 0.001 U/mg, respectively. Specific activity measurements were all statistically significant and agreed with the observed reduced abundance of WLP enzymes.

Interestingly, predicted BMC proteins encoded by one particular cluster were found to have enhanced abundances in this analysis for the Δ*agrD1D2* mutant (CLAU_1769, CLAU_1772, CLAU_1773, CLAU_1775 and CLAU_1785), along with 1,2-propanediol and two ethanolamine-utilising enzymes, PduL, EutQ and EutJ (CLAU_1771, CLAU_1774 and CLAU_1779) respectively. A second BMC cluster with enhanced abundances, represented another BMC associated protein together with further predicted 1,2-propanediol and ethanolamine utilising enzymes (CLAU_3206, CLAU_3202 and CLAU_3205). Finally, uncharacterised proteins CLAU_1786, 1780 and 1788 were noted for their enhanced abundances in the mutant, and proximity to BMC clusters. The first and third were related to PadR-type transcriptional regulators, involved in phenolic-acid stress^62,63^, the second uncharacterised protein shared high similarity with *C. ljungdahlii*’s ethanolamine utilisation cobalamin adenosyl transferase (EutT), involved in ethanolamine metabolism^64^. Phosphate acetyltransferase (Pta), CLAU_3274, involved in acetate formation was expected to be in low abundance in the Δ*agrD1D2* mutant, but interestingly did not appear to be significantly differentially regulated between the mutant or WT. Other notable fold differences in protein abundance were identified in several transcriptional regulators (Supplementary information, Table S6), but do not directly link to the observed ethanol/acetate and gas-assimilation phenotypes in Δ*agrD1D2* mutants.

## Discussion

Our understanding of clostridial cell-to-cell communication systems is still limited although several common roles have been characterised. Agr-type QS systems are known to influence sporulation in the few species that have been studied^11,50,52^ and in *C. botulinum* and *C. perfringens* they also contribute to the regulation of toxin production^51,54^. In two industrially relevant clostridia, *C. acetobutylicum* and *C. saccharoperbutylacetonicum* RRNPP-type systems are involved in the control of solvent metabolism and sporulation^9,10^. As will be argued below, the Agr QS system of the acetogen *C. autoethanogenum* may plausibly form part of a resource management mechanism through evaluating population density with respect to environmental conditions that include carbon source availability. Here we have shown that complete removal of both *agrD* signalling genes resulted in striking changes in both fermentative profiles and gas assimilation capabilities of the Δ*agrD1D2* mutant. Moreover, notable fold differences were observed in comparative proteome analysis between the Δ*agrD1D2* mutant and WT that included several key WLP related enzymes and a specific cluster of alcohol and aldehyde dehydrogenases.

Initial observations showed that single knockout *agrD* mutants did not exhibit any discernible phenotypic differences with respect to the WT. Full length AgrD1 and AgrD2 share an overall identity of 70.2% but are predicted to be processed into unique cyclic structures that are presumably recognised by distinct AgrC receptors. This raises questions as to why two relatively similar peptide signals exist and what possible function each serves. Further insight can be derived from supplementary transcriptomic data from the study done by Marcellin et al. revealing that the RNA-seq transcript counts of *agrD1* were fourfold higher in fructose-grown conditions (48.0 vs. 11.7), and sevenfold more in autotrophic conditions (162.4 vs. 22.2) than *agrD2*^34^. These different expressions can be speculated to act in a combinatorial mode of function, meaning that the two separate signalling peptides function separately but simultaneously, in cultures with high population density, cell diffusion and fluctuating mass transfer^65^. This can effectively improve an organism’s ability to dynamically adapt and prepare in ever-changing environments through precise gene expression mediation^65,66^. An acetogen able to mediate its WLP genes during specific growth conditions may spare valuable ATP from unnecessary protein synthesis during heterotrophic growth^67^. For instance, by integrating information on population size and available organic carbon sources, *C. autoethanogenum* may be able to determine more precisely when to increase costly expression of WLP enzymes, before organic substrates are depleted. Regarding this study, it can be assumed that disabling only either one of these AgrD AIPs merely reduces this spectrum, leaving the Agr system largely functional. It was also reasoned that AgrD1 and AgrD2 AIPs must be processed using the same AgrB1-mediator encoded by the single *agrB1* gene present within the genome, implying that AgrD2 peptides rely on expression of System 1. However, AgrD AIP detection is likely performed separately using either AgrC receptor from each respective *agr* operon (Fig. 1). Whether intracellular signalling pathways converge to a single, yet unidentified AgrA response regulator, or to the Spo0E-like regulator encoded downstream of *agrB1*, can be only speculated. A single AgrA response regulator could explain the absence of a clear phenotype in each single knockout *agrD* mutant by possibly allowing either AgrD AIP to compensate for the other missing AIP. Alternatively, regulatory feedback loops might lead to increased production of one of the AIPs, if the other one is lacking.

As neither single knockout mutant showed discernible phenotypic differences*,* efforts were focused to restore to a WT-like phenotype in the Δ*agrD1D2* mutant with each *agrD* gene. Figure 4 showed that this restoration was partially successful with acetate, ethanol titres and gas re-assimilation for the Δ*agrD1D2* + *D1comp* strain, but not for Δ*agrD1D2* + *D2comp* strain. This partial complementation may be explained by changes to the respective *agrD* expression profiles and levels^34^, given that complementing *agrD* genes were expressed via the *pyrE* operon promoter instead of their respective native promoters. Studies examining the Agr system of *C. acetobutylicum* demonstrated that mutagenic disruption of *agrA, agrB1* and *agrC* in this organism resulted in a reduction of heat-resistant endospores and loss of granulose storage compounds^11^. *C. autoethanogenum*, however, although a reported spore-former^22^, was not observed to produce spores under the conditions employed in this study (see “Materials and methods”), and granulose storage homologs are not present in its genome, and thus could not be used as additional phenotypic markers^68^. Phenotypic characterisation of the generated Δ*agrD1D2* mutants thus primarily concentrated on fermentation profiling and autotrophic growth on CO.

With respect to WT and mutant fermentation metabolism, we reproducibly observed high ethanol titres for the three independently generated Δ*agrD1D2* mutant, along with accumulated CO_2_ headspace pressures. A similarly high ethanol output was noted in Liew et al.’s study after CODH subunits, encoded by *acsA* (CLAU_1578-79), were genetically disrupted, resulting in a twofold ethanol increase, abolished acetate production, near-absent autotrophic growth and approx. fourfold accumulated CO_2_ headspace pressure^44^. The increased ethanol was reasoned to be the result of ethanol generation serving as the primary electron sink for the surplus reducing equivalents generated during glycolysis that could not be utilised by CODH/ACS. The similarities in results were intriguing but it must also be noted that Δ*agrD1D2*’s heterotrophic growth on fructose was not affected unlike the Δ*acsA* mutant which exhibited 61% lower stationary phase OD compared to the WT^44^. This distinction may raise an important clue as to how CODH/ACS and other key WLP enzymes are regulated as noted in previous studies, with transcriptomic data revealing CODH/ACS having relatively high transcript counts, even during heterotrophic conditions^34^. Therefore, it can be assumed that the Δa*grD1D2*’s lack of peptide signalling capability prevents it from accurately mediating *when* to express CODH/ACS and potentially other components of the WLP pathway, allowing for unhindered growth on sugar but with a reduced capacity to fully re-assimilate CO_2_. This phenomenon was further studied by growing Δa*grD1D2* autotrophically (Fig. 3 B-D), with initial growth considerably lower to that of the WT. Only after stepwise adaptation was the Δa*grD1D2* mutant eventually restored to WT growth and product titre levels.

Comparison of Δa*grD1D2*’s proteome to that of the WT supported observations of diminished CO_2_ assimilation capacity with the most negative fold differences found in CODH/ACS subunits, along with several hydrogenase subunits that associated to the WLP, as well as the formate dehydrogenase alpha subunit (CLAU_2713). Interestingly yet counterintuitively, a cluster of ethanol-forming enzymes were also noted to be downregulated, some of which are expressed during autotrophic growth^34^, and involved in acetate reduction to ethanol via AORs (CLAU_0089 and 0099)^69^. Conversely, the most positive fold changes included several putative aldehyde and alcohol dehydrogenase enzymes that may explain the high ethanol yields. Further, the highest overall positive fold changes belong to BMC clusters outside of the WLP. Although unexpected, this observation is perhaps unsurprising as BMC located enzymes sequester and metabolise toxic intermediary compounds and are reasoned to be concomitantly expressed with alcohol metabolism in other species^70–72^. Regarding our proteomics approach, the iTRAQ method employed in this study has its own limitations resulting from complex bacterial lysate mixtures, that may result in underestimating protein abundance^73,74^. Ultimately, this analysis was used as a comparative indicator of direction of positive or negative protein fold changes. To independently verify, we employed three enzyme assays that targeted the most negative and positive fold changes from our proteomics data (with the exception for BMCs). These assays confirmed Δa*grD1D2*’s lower activity of CODH and hydrogenases, and slightly higher activity of alcohol dehydrogenases, and agreed with the Δa*grD1D2* mutant’s phenotype.

To date, no studies have examined the role of QS in syngas-utilising acetogens despite complete and partial Agr systems being conserved across several industrially important species. Previous studies have established that these systems serve crucial functions in non-pathogenic *Clostridium* sp., such as the well-studied solventogen, *C. acetobutylicum*^10,11^. These studies emphasise the need of examining QS systems in industrially relevant biocatalysts. QS is a means of cell-to-cell signalling that ultimately aids in the organism’s survival. This system can be triggered through nearby stressors that allow organisms to metabolically prepare and acclimate to changing environments^75–77^. Through this study it can be reasoned that *C. autoethanogenum*’s Agr QS system plays the critical role of sensing population density in nature, allowing for prompt upregulation of enzymes required for C1 gas metabolism, well before energy-rich sugar sources are exhausted. It appears that many acetogens employ the WLP as an electron sink for redox balancing when growing with other organic compounds^42^ and hence it was interesting to see that in the complete absence of Agr QS, reducing equivalents generated during fructose oxidation appear to be channelled into ethanol formation. It can be reasoned that two differentially expressed^34^ AgrD peptides enable *C. autoethanogenum* to accurately monitor its environment and mediate the use of reducing equivalents for CO_2_ reduction through appropriate expression of the necessary WLP enzymes exactly when needed.

## Materials and methods

### Bacterial strains and growth conditions

Laboratory stocks of *C. autoethanogenum* DSM 10061 used for this study were purchased from Deutsche Sammlung von Mikroorganismen und Zellkulturen (DSMZ) GmbH, Braunschweig, Germany, (see Supplementary information, Table S1 for full list of strains). *Escherichia coli* One Shot® TOP10 used in plasmid construction, cloning and conjugation, was grown in LB medium or 1.5% solid agar LB plates. Agar LB plates were incubated at 37 °C, and liquid cultures shaken at 225 RPM at 37 °C with antibiotics where appropriate. Antibiotics and working concentrations used in this study were: chloramphenicol (25 μg/mL), thiamphenicol (7.5 μg/mL), spectinomycin (100 μg/mL) and d-cycloserine (250 μg/mL).

*Clostridium autoethanogenum* genetic engineering was performed in an anaerobic workstation (Don Whitley Scientific, Yorkshire, UK) at 37 °C. For general cultivation methods, YTF medium was used as described previously^68^. To screen for *pyrE*-deficient and uracil auxotroph mutants, semi-defined minimal medium (SDMM) that was similar to YTF but used per litre: 7.2 g d-fructose, 20 g 2-(N-morpholino)ethanesulfonic acid (MES) buffer and 10 g casein amino acids in place of yeast extract and tryptone.

For growth and fermentation analysis, experiments took place in 250 mL serum flasks containing American Type Culture Collection (ATCC) PETC 1754 medium, with the following additions and modifications: 20 g/L MES and 1.5 g/L casein amino acids in place of yeast extract. Serum flasks filled with medium inside of an anaerobic chamber (COY laboratory products, USA) with a gas atmosphere of 85% N_2_, 10% CO_2_ and 5% H_2_. After filling, flasks were sealed with rubber stoppers, crimped, and autoclaved. After autoclaving, 1 mL of 4% (w/v) L-cysteine was added to each serum flask. For sugar-based experiments, 90 mL of medium was dispensed into each flask with D-fructose added to a concentration of 50 mM post autoclave and placed into a New Brunswick Innova 44 shaking incubator (Eppendorf) set to 225 rpm, at 37 °C. Flasks were sealed with butyl rubber stoppers preventing any gas exchange. Analysis of *C. autoethanogenum* Δ*agrD* mutants using D-fructose used three to four technical replicates, that included a WT control, and up to six biological replicates. Starting OD_660_ was approx. 0.01 and flasks were usually cultured for up to 10 days with 10–12 sampling points. For gas-grown cultures, serum flasks were filled with 50 mL PETC, pressurised with 200 kPa CO and stored horizontally at 37 °C in a static incubator. Headspace pressure was determined via a needle attached to a handheld pressure gauge (Omega Engineering, USA). Experiments began with a starting OD_660_ of 0.01 with 2 biological replicates and were cultured for up to 30 days. Samples were taken approximately every 3–5 days. For all experiments growth was measured by measuring optical density at 660 nM (OD_660_) using a Jenway 7300 spectrophotometer (Bibby Scientific, UK).

### Detection of spores

To determine sporulation activity of WT *C. autoethanogenum*, stocks were grown in 3 mL YTF in triplicate for up to 5 days. Two mL from each culture was spun down, washed, and resuspended in 400 μL of anaerobic PBS. This mixture was split into equal aliquots, one acting as a control, the other was heat shocked at 80 °C for 10 min using a SensoQuest PCR machine (SensoQuest GmbH, Germany). The experimental and control triplicates were serially diluted ranging from 10^0^ to 10^–2^ and plated onto YTF plates, colonies appeared after 4–5 days. *C. acetobuytlicum* was used as a control and was grown on clostridial basal medium (CBM). *C. acetobuytlicum* was subjected to identical heat-shock procedures as *C. autoethanogenum* and plated on either CBM or YTF agar plates respectively. Samples were observed using under a phase-contrast filter of a Nikon Eclipse Ci-E microscope (Nikon Instruments Europe B.V., UK). Visualising the presence of spores was done using Schaeffer and Fulton Spore Staining Kit (Sigma Aldrich, UK) as per manufacturer’s instructions.

### DNA and plasmid manipulations

Routine genomic DNA extraction used in PCR diagnostics used PureLink Genomic DNA extraction (Invitrogen/ThermoFischer). Plasmid purification from *E. coli* was performed using GenElute Plasmid Miniprep kits (Sigma Aldrich, UK). All purified DNA fragments and plasmids were quantified using NanoDrop Lite (Thermo Scientific, UK). High-quality, proof-read amplification of DNA fragments, for short-length sequencing and cloning used Phusion High-Fidelity DNA Polymerase (NEB, UK). Routine screening and analytical PCR procedures used DreamTaq Green DNA polymerase (Thermo Scientific, UK). Primers were synthesized by Sigma-Aldrich or MWG Eurofins. Sanger sequencing of plasmids and amplicons was carried out by Source Bioscience PLC (UK). All primers used in this study are listed in Supplementary information, Table S2.

### Creation of Allelic coupled-exchange plasmids

All plasmids were based on the pMTL80000-series Modular Plasmid System^78^, specifically pMTL84151. A full list of created plasmids is shown in Table S3. For all targeted genes, inactivation was achieved using an asymmetrical homology arm design as outlined by Minton et al., allowing single and double crossover events to occur in a preferred order^79^. Plasmid pMTL84151_ΔpyrE was constructed to generate a 3’ truncated version of the *C. autoethanogenum pyrE* gene thereby creating the *pyrE-negative* parent strain used for all further mutations. Its design followed the principles outlined previously^79^; it carried a non-homologous *C. acetobutylicum pyrE* gene serving as a counter-selection marker for single cross-over event screening, and an allelic-exchange cassette consisting of the left homology arm (LHA) (303 bp), the right homology arm (RHA) (1219 bp) of *C. autoethanogenum*’s *pyrE* and a lacZα fragment (264 bp) in between. Primer pairs used to amplify the LHA and RHA were pyrE_FSP, see Table S2 for list of primer sequences.

Plasmids pMTL84151_0816 and pMTL84151_3094 were designed to inactivate Δa*grD1* or Δ*agrD2,* respectively and both were constructed using the original pMTL84151 backbone^60^. LHA and RHA amplicons were generated by PCR, using two sets of primer pairs for each homology arm as follows: For CLAU_0816, primer set; 0816_LHA_F + R and 0816_RHA_F + R was used. Similarly, primer pair sets for and pairs for *agrD2*; 3094_LHA_F + R and 3094_RHA_F + R. Each set was combined using splice-overlapping extension PCR (SOE-PCR) to produce inserts of 1,270 bp and 1,437 bp inserts*,* respectively. Both inserts were subsequently ligated into the pMTL84151 backbone producing plasmids pMTL84151_0816 and pMTL84151_3094. Each in-frame deletion (IDF) region was designed to remove approx. 80% of the gene.

Plasmid pMTLCH20^60^ was used to repair the *pyrE* defect in the generated *agrD* mutants, again as outlined by Minton et al., the insert of this plasmid encoded an intact *pyrE* gene, generated by combining LHA and RHA of 524 bp and 1212 bp, respectively^79^. Two derivatives of this plasmid, complementation vectors pMTLCH20-D1 and pMTLCH20-D2, were used to combine repair of the *pyrE* gene with downstream integration of intact *agrD1* and *agrD2* genes, respectively. Primer pairs 0816_Comp_F + R for *agrD1*, and 3094_Comp_F + R for *agrD2* were used to amplify each *agrD* region respectively and the obtained PCR products were similarly digest and ligated into pMTLCH20.

### Plasmid conjugation

Plasmids were introduced into *C. autoethanogenum* through previously established methods^80^, using *E. coli* CA434 as a conjugation donor strain. Selected plasmids were first electroporated into the donor strain, performed in a 0.2 cm wide electroporation cuvette (Biorad, UK) at 2.50 kV. After conjugation, YTF agar plates containing D-cycloserine and thiamphenicol were used to counter-select against *E. coli* CA434. Colonies of conjugated *C. autoethanogenum* typically appeared after 4–5 days. Larger, fast-growing colonies, assumed to be successful transformants, were picked and re-streaked onto fresh selective YTF containing the same antibiotics. Plasmids were introduced into *C. autoethanogenum* through previously established methods^80^, using *E. coli* CA434 as a conjugation donor strain. Selected plasmids were first electroporated into the donor strain, performed in a 0.2 cm wide electroporation cuvette (Biorad, UK). Electroporation was performed using a MicroPulser electroporator (Bio-Rad, UK) at 2.50 kV for 5 mS. After conjugation, YTF agar plates containing D-cycloserine (250 μg/mL) and thiamphenicol (25 μg/mL) were used to counter-select against *E. coli* CA434 and presence of the plasmid in *C. autoethanogenum* recipient cells, respectively. Colonies of plasmid-carrying *C. autoethanogenum* typically appeared after 4–5 days. Larger, fast-growing colonies, assumed to be successful transformants, were picked and re-streaked onto fresh selective YTF containing the same antibiotics.

### Creation of a *C. autoethanogenum*_Δ*pyrE* mutant

First, a *pyrE* deficient strain of *C. autoethanogenum* was created which would serve as a host for later *agrD* IFD mutations with positive and negative selection markers^57^. To do this, *C. autoethanogenum* WT was conjugated with *E. coli* CA434 pMTL84151_ΔpyrE. Following selection on thiamphenicol, the initial, large *C. autoethanogenum* colonies resulting from this conjugation were picked and placed into liquid YTF containing thiamphenicol and grown for 3 days. Three PCRs were used to screen for pMTL84151_ΔpyrE singe-cross over integrants: first, using flanking primer pyrE_RSP with internal primer 84151_F, second, using flanking primer pyrE_FSP with internal primer 84151_R. The third reaction used both flanking *pyrE* primers, covering the entire integration region to reveal no bands as the amplicon was too large. Possible amplicons in the third reaction suggested plasmid integration was contaminated with the presence of WT *pyrE* and required repetition to obtain a pure single-cross integrant. Confirmed single-cross integrants were plated onto YTF plates containing 1 mg/mL of 5-fluoroorotic acid (FOA) and 20 μg/mL uracil to select for the double crossover event. FOA-resistant colonies appearing after 3 days were picked and replica plated onto two sets of SDMM agar plates with and without 20 μg/mL uracil. Colonies that grew exclusively on uracil supplemented SDMM plates were re-streaked again onto SDMM + /- uracil plates. Confirmed colonies were cultured in YTF for gDNA extraction and storage at -80 °C. Extracted gDNA was to amplify the now truncated *pyrE* region using flanking primers pyrE_FSP and pyrE_RSP. Amplicons were purified and Sanger sequenced for confirmation. Plasmid loss was determined through streaking newly created Δ*pyrE* strains onto YTF plates containing thiamphenicol. Lack of growth indicated pMTL84151_ΔpyrE plasmid loss.

### Creation of single and double knockout *agrD* mutants

IFD plasmids pMTL84151_0816 and pMTL84151_3094 were conjugated separately into *C. autoethanogenum_*Δ*pyrE* to produce the respective single knock-out mutants. After the second round of selection, carried out as described above for ∆*pyrE*, large colonies were verified for single cross integration using. Right and left homology arm flanking primers for each target gene (0816_SCR_F + R and 3094_SCR_F + R) were paired with pMTL84151 internal primers 84151_F and 84151_R in three separate PCRs. Single-cross integrants were isolated and plated onto FOA YTF plates and grown for 3 days to promote double crossovers. Visible colonies were replica plated onto SDMM +/− uracil plates and grown for a further 4–5 days. To screen for successful target gene truncation, colonies that grew exclusively on uracil plates were screened using respective 0816 and 3094 flanking primers. Sanger sequencing confirmed in-frame truncation and mutants Δ*agrD1_*Δ*pyrE* and Δ*agrD2_*Δ*pyrE* were created.

For the creation of the Δ*agrD1D2* double mutants, strains Δ*agrD1_*Δ*pyrE* or Δ*agrD2_*Δ*pyrE* were used and were conjugated with either pMTL84151_3094 or pMTL84151_0816, respectively. This created three independent mutants: Δ*agrD1D2_*Δ*pyrE*, Δ*agrD1D2_1* Δ*pyrE* and Δ*agrD1D2_2* Δ*pyrE*.

### Restoration and complementation techniques

Restoration of the *pyrE* locus in Δ*agrD1_*Δ*pyrE,* Δ*agrD2_*Δ*pyrE* and Δ*agrD1D2_2* Δ*pyrE* mutants used plasmid pMTLCH20 and followed allelic-couple exchange methods outlined earlier^61^. This plasmid was first conjugated into Δ*pyrE* mutant strains as described above. Fast-growing colonies were streaked directly onto SDMM +/− uracil plates and grown for 4 days. Colonies growing without uracil supplementation were selected and primers, pyrE_FSP and pyrE_RSP were used for PCR screening and Sanger sequencing to confirm *pyrE* restoration. A 264 bp LacZα marker was incorporated into *pyrE* restored mutants.

Complementation of the Δ*agrD1D2* double mutant followed the same procedure but utilised pMTLCH20 derivatives pMTLCH20-D1 and pMTLCH20-D2, respectively. Conjugation took place as described previously and successful transformants were replica plated onto SDMM +/− uracil plates and grown for 4 days. Colonies growing without uracil supplementation were confirmed for *pyrE* locus restoration and insertion of respective *agrD* genes. PCR screening using pyrE_FSP and pyrE_RSP flanking primer and Sanger sequencing of the generated amplicons confirmed creation of the complemented mutants, Δ*agrD1D2* + *D1comp* and Δ*agrD1D2* + *D2comp*.

### Genome sequencing

To confirm intended mutations, genome sequencing was performed on all single and double knock-out mutants that were *pyrE* corrected, using Illumina next generation sequencing (Microbes NG, Birmingham). Raw genomic data was processed and assembled using CLC Genomics Workbench (v. 8.0.2), (Qiagen, Denmark) and were mapped against the Humphreys *et. al. C. autoethanogenum* DSM 10,061 genome^36^.

### Gas chromatography (GC)

Supernatant samples of 500 μL were pipetted into 1.5 mL tubes and acidified with 5 μL of 10 M H_2_SO_4_. Samples were vortexed at 21,000×*g* for 1 min, and 500 μL of propyl propionate containing 50 mM valeric acid was added as an internal standard and samples were vortexed once more. The extraction mixture was spun down again to remove denatured proteins. Samples then separated into a biphasic solution, from which 300 μL of the organic upper phase was removed and pipetted into a 300 μL glass insert that was placed inside a 2 mL brown sampling vial. Analysis of metabolites from prepared samples used a Focus gas chromatograph (Thermo Fischer Scientific) with a 30 m TRACE TR-FFAP column (Thermo Fischer Scientific) with 0.25 mm internal diameter. A flame ionisation detector was maintained with 350 mL/min compressed air, 35 mL/min H_2_ and 30 mL/min N_2_. The H_2_ carrier gas was supplied at 0.8 mL/min. Injector inlet temperature was maintained at 240 °C and detector temperature was set to 270 °C. For resolution of peaks the following column program was used: after injection, temperature was held at 40 °C for 2 min, then ramped to 150 °C at a rate of 80 °C/min, and finally to 210 °C for 1 min.

### Gas headspace analysis

Serum flask headspace composition was measured with a TRACE gas chromatograph (Thermo Fischer Scientific). 1 mL of gas was removed from serum flasks with gas-tight syringes and manually injected. A 30 m TRACE TR-FFAP column (Thermo Fischer Scientific) with a 0.25 mm internal diameter was used with the following program for peak resolution: After injection, temperature was held at 40 °C for 3 min, then ramped to 250 °C at a rate of 30 °C/min, the duration of each run took 10.7 min. Standards for gas analysis, comprised of 1% CO, 1% CO_2_, 1% H_2_, 97% N_2_.

### High performance liquid chromatography

Centrifuged supernatant samples were mixed in a 1:1 volume ratio with internal standard and mobile phase solution (80 mM valeric acid in 0.005 M H_2_SO_4_). Protein and cell debris were removed from samples by centrifugation and syringe filtration. Samples were measured using a Dionex HPX-Ulti-Mate 3000 HPLC system (Thermo Fischer Scientific, UK). 20 μL volumes were injected into a Bi-Rad Aminex 87H (Biorad UK) column, maintained at 35 °C, with a 0.005 M H_2_SO_4_ mobile phase at a flow rate of 0.5 mL/min. Peaks were detected through a refractive index and diode array detector at UV 210 nm.

### Preparation of samples for proteomic analysis

Cultures of WT *C. autoethanogenum* and *C. autoethanogenum*_Δ*agrD1D2* were grown in triplicate in 250 mL serum flasks containing PETC medium with 50 mM D-fructose. Cultures were harvested at approx. OD_660_ 1.0, after approx. 3–4 days and centrifuged at 3000×*g* for 5 min and supernatant was removed. The cell pellets were stored on ice and washed twice with PBS and once with protein extraction buffer, triethyl ammonium bicarbonate (TEAB), 0.5 M pH 8.5 (Sigma Aldrich). Samples were then flash frozen in liquid nitrogen and sent to the University of Sheffield to be analysed by Dr Mahendra Raut using LC–MS/MS-based iTRAQ methods (4-plex reagents isobaric and tags for relative and absolute quantitation). See Supplementary information, Sect. 2 for full protocol.

### Crude lysate preparation for enzymatic assays

*Clostridium autoethanogenum* WT and Δ*agrD1D2* cells were grown in duplicate in 750 mL serum flasks containing 400 mL PETC with 50 mM D-fructose. Cells were harvested at early to mid-exponential phase at OD_660_ 0.4–0.45 in an anaerobic chamber (COY laboratory products, USA). Cultures were centrifuged at 3,000 × g for 5 min and following removal of the pellets of each strain were flash frozen in liquid N_2_ and stored at -80 °C. Cell pellets were resuspended thoroughly in a combination of 750 μL of BugBuster protein extraction reagent (Merck Millipore, United States) with 750 μL 50 mM TRIS–HCl and 0.5 mM MgCl_2_, pH 7.0, this mixture was prepared anaerobically. 10 mg/mL of lysozyme, and 1 mg/mL of DNase (Sigma Aldrich) was added to the mixtures and gently aspirated. The lysis mixture was placed into a pre-evacuated 15 mL Hungate tube and sealed. The tube was placed onto a rocker at room temperature at 50 RPM for one hour. Tubes were placed in the anaerobic chamber and each tube’s mixture was pipetted into a 2 mL cryovial (Sarstedt, Germany) and spun at 20,000×*g* at 4 °C for 20 min. The clear supernatant was injected into a 5 mL serum flask vacuum filled with pure N_2_ and stored on ice ready for analysis. For full specific activity measurement methods followed published procedures^35,81^, for full methodology see Supplementary information, Sect. 2.

### Data analysis and presentations

Analytics data was collected using Chromeleon 7.2 Chromatography Data System (Thermo Fisher Scientific, UK). Data was compiled using Microsoft Excel 2010 which compared samples using used two-tailed, unpaired, parametric student's T-test. Graphical representation used GraphPad Prism 7.

## Supplementary Information

Supplementary Information.
