# Supplementary material for: Agr Quorum Sensing influences the Wood-Ljungdahl pathway in Clostridium autoethanogenum
Source: Sci Rep. 2022 Jan 10;12:411. doi: 10.1038/s41598-021-03999-x (PMC8748961; doi:10.1038/s41598-021-03999-x)
Supplement: Supplementary file 1 — Supplementary Information. [file 41598_2021_3999_MOESM1_ESM.docx]

**Supplementary Information**

**Agr Quorum Sensing influences the Wood-Ljungdahl pathway in *Clostridium autoethanogenum***

Pawel Piatek^1^, Christopher Humphries^2^, Mahendra P. Raut^3^, Phillip C. Wright^4^, Sean Simpson^5^, Michael Köpke^5^, Nigel P. Minton^2^, Klaus Winzer^2*^

1. Department of Biotechnology and Nanomedicine, SINTEF Industry, 7465 Trondheim, Norway
2. BBSRC/EPSRC Synthetic Biology Research Centre (SBRC), School of Life Sciences, University Park, The University of Nottingham, Nottingham, United Kingdom
3. The ChELSI Institute, Department of Chemical and Biological Engineering, University of Sheffield, Mappin Street, Sheffield S1 3JD, United Kingdom
4. University of Southampton, University Road, Southampton, SO17 1BJ, United Kingdom
5. LanzaTech Inc., 8045 Lamon Ave, Suite 400, Skokie, IL 60077, USA

*Corresponding author

**Section 1: Tables and Figures**

Table S1

| Strains used in this study: C. a. = *C. autoethanogenum*, and E. c. = *E. coli*; DSMZ = Deutsche Sammlung von Mikroorganismen und Zellkulturen (Braunschweig, Germany). | | |
| --- | --- | --- |
| Strains | **Description** | **Source** |
| *C. a.* JA1-1 DSM 10061 | Wild type Strain | DSMZ |
| *C. a.* Δ*pyrE* | *pyrE* gene deficient strain for allele coupled exchange | This study |
| *C. a.* Δ*agrD1* | *agrD1* deficient strain | This study |
| *C. a.* Δ*agrD2* | *agrD2* deficient strain | This study |
| *C. a.* Δ*agrD1D2* | *agrD1* and *agrD2* deficient strain | This study |
| *C. a.* Δ*agrD1D2_1* | *agrD1* and *agrD2* deficient strain; independently obtained | This study |
| *C. a.* Δ*agrD1D2_2* | *agrD1* and *agrD2* deficient strain; independently obtained | This study |
| *C. a.* Δ*agrD1* Δ*pyrE* | *agrD1* and *pyrE* truncated strain | This study |
| *C. a.* Δ*agrD2* Δ*pyrE* | *agrD1* and *pyrE* truncated strain | This study |
| *C. a.* Δ*agrD1D2* Δ*pyrE* | *agrD1*, *agrD2* deficient strain and *pyrE* truncated strain | This study |
| *C. a.* Δ*agrD1D2_1* Δ*pyrE* | *agrD1*, *agrD2* deficient strain and *pyrE* truncated strain | This study |
| *C. a.* Δ*agrD1D2_2* Δ*pyrE* | *agrD1*, *agrD2* deficient strain and *pyrE* truncated strain | This study |
| *C. a.* Δ*agrD1D2+D1comp* | Double knockout mutant with *agrD1* complementation down-stream of *pyrE* | This study |
| *C. a.* Δ*agrD1D2+D2comp* | Double knockout mutant with *agrD2* complementation down-stream of *pyrE* | This study |
| *E. c.* One Shot® TOP10 | General cloning chemically competent *E. coli* strain | Thermofischer |
| *E. c.* CA434 | *E. coli* HB101 strain carrying R702 conjugative plasmid, used for all conjugations | Dr. Chris Humphreys (Uni. Of Nottingham) |

Table S2:

| Primers used in this study: Underlined sequences represent restriction sites | | |
| --- | --- | --- |
| Oligonucleotides | **Sequence 5’-3'** | **Description** |
| pMTL_84151 screening primers | | |
| 84151_F | GAGCAAGGCAAGACC | IFD plasmid screening |
| 84151_R | GCTAAAAATCTATTTATATTTCACC |  |
| In-frame deletion plasmid construction primers | | |
| 0816_LHA_F | ATATATCCGCGGGGTACTTTAAAAAAATCATCC | SOE-PCR primers for IFD *agrD1* plasmids |
| 0816_LHA_R | TTCATCAGGATTTTTCATTTCATACACTCC |  |
| 0816_RHA_F | AATGAAAAATCCTGATGAATTCTTAAAGTAA |  |
| 0816_RHA_R | ATATATGGCGCGCCAGATAAGACCTTCTTTTTCG |  |
| 3094_LHA_F | ATATATCCGCGGCCAGATAATTTGTTTTTAGC | SOE-PCR primers for IFD *agrD2* plasmids |
| 3094_LHA_R | TTCATCAGGATTTTTCATTTTTCATATACC |  |
| 3094_RHA_F | AATGAAAAATCCTGATGAACTTTTGAAATAA |  |
| 3094_RHA_R | ATATATGGCGCGCCGCAAAATACTAAGATAGTAACAC |  |
| In-frame deletion mutant screening primer | | |
| 0816_SCR_F | CCTTATTTCTAAAATTATGG | Flanking primers screening *agrD1* |
| 0816_SCR_R | GGTCTGTTTTTCCTTTAAGC |  |
| 3094_SCR_F | GGCTATTTACAACATTGTCC | Flanking primers screening *agrD2* |
| 3094_SCR_R | CATTTACAAGTGTTTGTTTTTCC |  |
| Complementation plasmid construction primers | | |
| 0816_Comp_F | ATATGCGGCCGCGCTATATAATTACAAAATTTTTACTAAAGG | Complementation fragment construction primers |
| 0816_Comp_R | ATATGCTAGCGCAGTATTTGTAAAAAAACTAAG |  |
| 3094_Comp_F | ATATGCGGCCGCGAATTTATAAATTAAAACATTATAGGAGG |  |
| 3094_Comp_R | ATATGCTAGCACCTAAGTATTTGTAAAAGAC |  |
| Universal screening primers | | |
| M13F | TGTAAAACGACGGCCAGT | *pyrE* restoration and deletion screening primer |
| pyrE_FSP | CATCAAAGCTATACTATTTTCCGTATTTACATTTGGG | Flanking primers screening *pyrE* |
| pyrE_RSP | GTTTGCAGTACCTACTTGAATTGCATAAGCTC |  |

Table S3:

| Plasmids used in this study | | |
| --- | --- | --- |
| Plasmid | **Description** | **Source** |
| pMTL84151_ΔpyrE | Used in creation of the *C. autoethanogenum* Δ*pyrE* strain | Anne Henstra (Uni. Of Nottingham) |
| pMTLCH20 | *pyrE* correction cassette and gene knock-in plasmid | Dr. Chris Humphreys (Uni. Of Nottingham) |
| pMTL84151_0816 | In-frame deletion plasmid for *agrD1* | This study |
| pMTL84151_3094 | In-frame deletion plasmid for *agrD2* | This study |
| pMTLCH20-D1 | Chromosomal complementation plasmid of *agrD1* | This study |
| pMTLCH20-D2 | Chromosomal complementation plasmid of *agrD2* | This study |

Table S4:

| Comparison of Agr proteins from *C. autoethanogenum* and related acetogens. Numbers represent percent identity. Absent homologs are represented by a dash “-“. | | | | | | | |
| --- | --- | --- | --- | --- | --- | --- | --- |
|  | **System 1** | | | | **System 2** | | **Genome Sequence** |
| Species | AgrB1 | AgrC1 | AgrD1 | Spo0E-like | AgrD2 | AgrC2 |  |
|  |  |  |  |  |  |  |  |
| *C. ljungdahlii* | 100 | 82 | 98 | 100 | 83.3 | 98 | (Köpke *et al.*, 2010) |
| *C. ragsdalei* | 92 | 79 | 77 | 91 | 79 | 82 | (Huhnke, Raymond L.; Lewis, Randy S.; Tanner, 2008) |
| *C. coskatii* | 98 | 79 | 79 | 100 | 77 | 81 | (Bengelsdorf *et al.*, 2016) |
| *C. scatologenes*^†^ | 73 | 64 | 51 | - | - | - | (Zhu *et al.*, 2015) |
| *C. drakei*^†^ | 73 | 64 | 53 | - | - | - | (Jeong *et al.*, 2014) |
|  |  |  |  |  |  |  |  |

^†^Both *C. scatologenes* and *C. drakei* possess a homolog of AgrD1, CSCA_2234 and B9W14_RS08495, respectively, that share 51 and 53% protein sequence identity with *C. autoethanogenum*'s AgrD1. They also possess a further three *agrD*-like genes with significantly lower protein sequence similarities when compared to AgrD1 and AgrD2 of *C. autoethanogenum*. These are CSCA_2180, CSCA_2292, and CSCA_2945 for *C. scatologenes* and B9W14_RS04890, B9W14_RS08250 and B9W14_RS08755 for *C. drakei.* CSCA_2292 and B9W14_RS08250 are also identical to an AgrD homolog present in *C. carboxidivorans* P7 (Ccar_RS26630).

Table S5:

| Conserved AgrD ring structures and putative AgrB recognition sites in related acetogens.  Locus tags present the following strains: CLAU: *C. autoethanogenum*, CLJU: *C. ljungdahlii*, CLRAG: *C. ragsdalei*, CSCA: *C. scatologenes*, B9W14: *C. drakei*, CLCOS: *C. coskatii* PTA-10522*.* Ring structure region = yellow highlight, a Pro-X-X-Pro motif = green highlight. Consensus symbols represent; *= fully conserved residue, : = conserved residue with strongly similar properties, .= conserved residue with weakly similar properties | |
| --- | --- |
| AgrD1 |  |
| CLAU_0816 | MKNLKKSLLSKTTKVVGSLSLFLAAIVIVPT STGGA YQPKCPDEFLK |
| CLJU_c28500 | MKNLKKSLLSKTMKVVGSLSLFLAAIVIVPT STGGA YQPKCPDEFLK |
| CLRAG_34580 | MKNLKKNFLNKSMKMVGYLSLFLAALVITPA SAGCG HQPQCPDELLK |
| CSCA_2234 | MMFFKKNFSKKVLKTLGSVCLFLGTLAIVPT SYASG QQPKCPDELLK |
| B9W14_RS08495 | MMFFKKNLSKKVLKTLGSVCLFLGTLAIVPT SYASG QQPKCPDELLK |
| CLCOS_10790 | MKNLKKTLLSKIMKVVGSISLFLAAIVITPT SLGLG YQPKCPKELLK  * :**.: .* * :* :.***.::.*.*: * . . **:**.*:** |
|  |  |
| AgrD2 |  |
| CLAU_3094 | MKNLKESVLKKSMKVVGCLSLFLEALVIVPA SAGCY HQPKCPDELLK |
| CLJU_c25570 | MKNLKESVLEKSMKVVGKLSLGLAEIVITPA SSGCA YQPKCPDELLK |
| CLRAG_03770 | MKNLKKSLLSKTMKAVGSLSLFLAAIVITPA SLGMG HQPKCPEDLLK |
| CLCOS_35050 | MKNLKESVLEKSMKVVGKLSLGLAEIVITPA SSGCA YQPKCPDELLK  *****:*:*.*:**.** *** * :**.** * * :*****::*** |
|  |  |

Figure S1:

**Figure S1. Workflow of *agrD* mutant creation in *C. autoethanogenum*:** Black arrows represent transformation via conjugation using plasmids with targeting knock-out cassettes; pMTL84151_0816 (Δ*agrD1*) and pMTL84151_3094 (Δ*agrD2*) respectively. Restoration of *pyrE* function was done using plasmid pMTLCH20. The step is represented through blue arrows. Finished, “*pyrE*-corrected” mutant strains are highlighted in bold and underlined. The final Δ*agrD1D2* strain is the original mutant produced from the parental Δ*pyrE* Δ*agrD1*. Mutants Δ*agrD1D2­_1+2* (Δ*agrD1D2­_1* and Δ*agrD1D2­_2*) are two additional strains independently made from the Δ*pyrE* Δ*agrD2* parental strain.


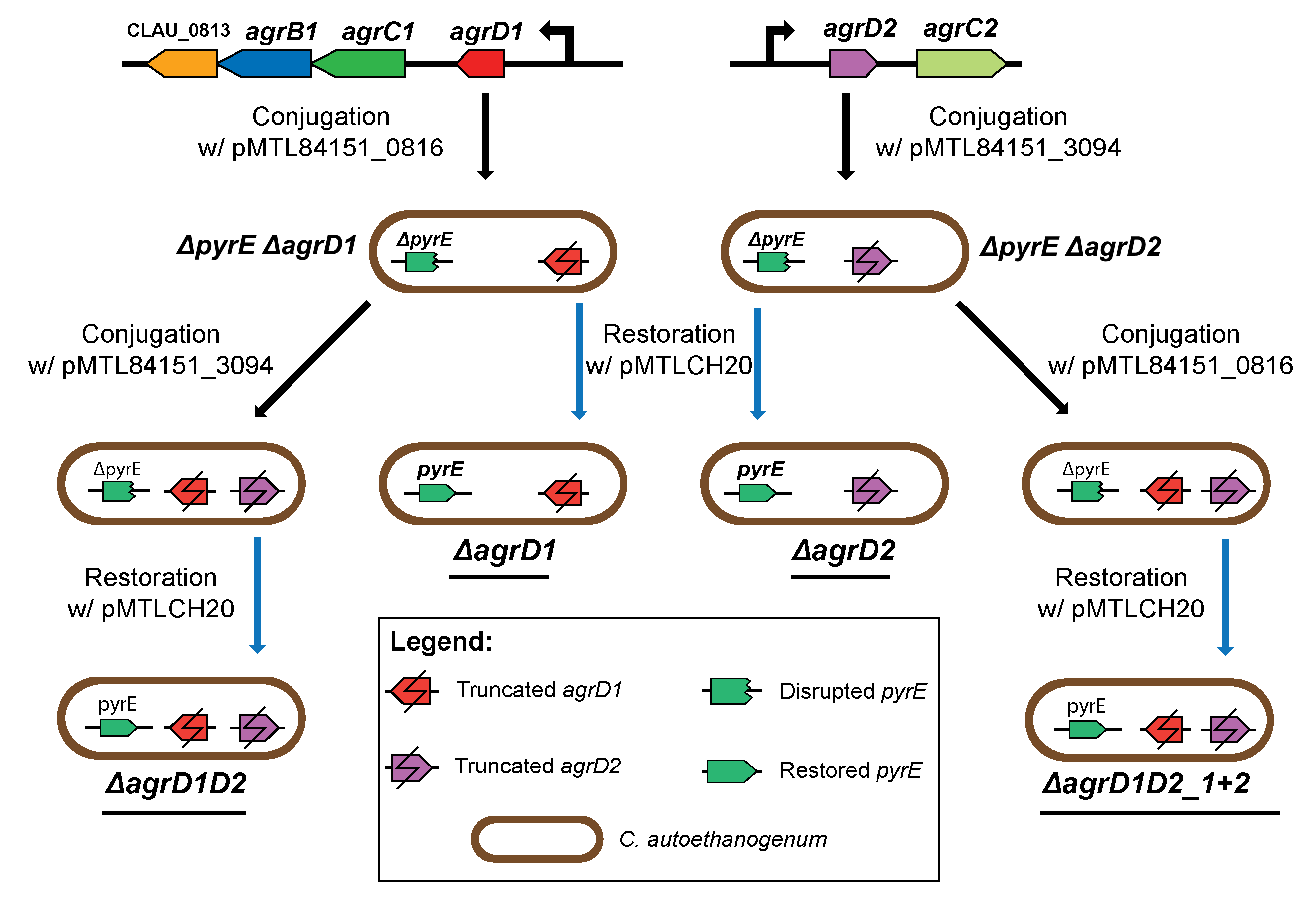


**Section 2: Protocols used in this study:**

Proteomics Analysis

Cell harvesting and protein extraction

Samples selected from each biological duplicate were labelled as follows: WT *C. autoethanogenum*, WT1 and WT2; *C. autoethanogenum* Δ*agrD1D2*, D1 and D2.

Thawed cells were resuspended in 600 µL lysis buffer (0.5 M TEAB containing 0.95 g L-1 Sodium dodesyl sulphate (SDS) and 5 μL protease inhibitor cocktail set II, pH 8.5) and 300 mg sterilized washed glass beads (425-600µm) were added. Cells were disrupted using bead beating (Disruptor Genie, Scientific Industries Ltd, USA) included 20 cycles for each for 2 min, with 1 min on ice between each run. Unbroken cell and debris were pelleted by centrifugation at 3,000 x g for 5 min. Supernatant-contained soluble proteins were transferred to new low-protein binding Eppendorf tubes (Eppendorf, Cambridge UK). This supernatant was further clarified by centrifugation at 17,000 x g for 45 min at 4 °C.

Proteins were acetone precipitated using a ratio of 1:4 (v/v) of sample to acetone. The mixture was incubated (approximately 16 hrs) overnight at -20 °C. Finally, protein pellets were re-dissolved in 0.5 M TEAB buffer containing 1 g/L RapiGest, a protein solubilising reagent (Waters, Milford, MA). Protein concentration was determined by Bradford assay according to the manufacturer’s protocol (Sigma Aldrich). Briefly, 20 µL of protein sample (1:20 dilution) was mixed with 980 µL of Bradford reagent, mixed and incubated for 10 min at room temperature. Intensity was recorded at 580 nm and concentration determined using a standard calibration curve obtained from standard BSA.

100 µg of each protein sample was dissolved in 0.5 M TEAB buffer (pH 8.5) containing 0.1 % (v/v) RapiGest and reduced with 1 µL of 50 mM Tris (2-carboxyethyl) phosphine hydrochloride (TCEP) at 60 °C for an hour. Subsequently samples were alkylated using 1 µL of 200 mM methyl methanethiosulfonate (MMTS). In the next step, samples were digested with a 1:50 (w/w) trypsin (Promega, UK) to protein ratio by overnight incubation for 16 hours at 37oC at constant shaking. The supernatant containing digested peptides was centrifuged at 13,000 x g for 5 min and collected supernatant was collected dried in vacuum concentrator (Scanvac; module speen 40, Lynge, Denmark).

iTRAQ labelling and Hypercarb fractionation

iTRAQ 4-plex labelling was performed on all biological phenotype according to the manufacturer’s protocol (8-plex iTRAQ reagent Multiplex kit, ABSciences, USA) as described previously (Raut *et al.*, 2016). Briefly, the relevant iTRAQ reagent re-suspended in a 50 µL of isopropyl alcohol and added carefully to respective sample tubes (i.e. WT1, WT2 labelled with 114, 115; D1, D2 labelled with 116, 117).

After labelling, samples were vortexed, spun down, and incubated for 2 hours at room temperature with gentle shaking. Next, the contents of all the labelled peptides were subsequently pooled into a 1.5 mL tube vortexed and spun down. Labelled samples were acidified with TFA (trifluroacetic acid, final concentration of solution 0.5 %) to precipitate out RapiGest. The supernatant was obtained by centrifugation at 17,000 x g for 5 min at 4 °C. Supernatant containing labelled peptides were dried using a vacuum concentrator (Scanvac; module speen 40, Lynge, Denmark) and kept at -20 ^o^C until further analysis.

iTRAQ labelled peptides were re-dissolved in 100 µL of Hypercarb buffer A (97% H_2_O, 3 % acetonitrile (ACN), 0.1 % TFA and submitted to UHPLC Ultimate 3000 RS (Dionex, Thermo, Hemel Hempstead, UK) coupled with a Hypercarb column (porous graphitic column; 7 µm particle size, 50 mm length, 2.1 mm diameter and 250 Å pore size, (Thermo Scientific, Waltham, MA, USA). Fractionation was carried out at flow rate of 0.2 mL/min at 240 nm. The elution of peptides was performed using a 55 min solvent gradient of buffer B (97 % ACN, 3% H_2_O, 0.1 % TFA). The 55 min gradient was set as 2 % B for 0 min, 2-10 % B for 5 min, 10-60 % for 30 min, 60-90 % B for 1 min, 90 % B for 6 min, 90-2 % B for 1 min and 2 % B for 12 min. Fractionation and chromatography was monitored through Chromeleon software (Dionex, Netherlands). A total of 18 fractions were collected for every 3 min and dried in a vacuum concentrator (Genevac Ltd Suffolk, UK). Out of 18 fractions, 12 fractions were selected for further LC-MS analysis (15-48 min).

Each fraction was re-dissolved in 20 µL reverse phase (RP) buffer A (3 % ACN, 0.1 % FA) and combined to obtained 6 fractions and subjected to further LC-MS/MS analysis. The fractions submitted to Q Exactive^TM^ Hybrid Quadrupole-Orbitrap^TM^mass spectrometer (Thermo Scientific, Bremen, Germany) coupled with an online UHPLC system (Ultimate 3000, Dionex, Surrey UK). 2 µL of each fraction was injected into the system, peptide separation was performed by PepMap RSLC column (C-18, 2 µm, 100Å, 75 µm x 50 cm) at a constant flow rate of 300 mL/min. A 105 min gradient was performed using RP buffer B (97 % ACN with 0.1 % FA) as follows: 3 % B for 0 min, 3 % B for 5 min, 3-10 % of B for 5 min, 10-50 % of B for 75 min, 50-90 % B for 1 min, 90 % for 4 min, 90-91 % for 1 min and finally 3 % of buffer B for 14 min. MS data was acquired with settings; for MS 60,000 resolution, AGC target 3e6, maximum IT 100ms and over scan range 100-1500 m/z. For MS/MS the following settings were selected. 15,000 resolution, AGC target 5e4, maximum IT 120 ms. Fifteen MS/MS were acquired per MS scan with normalised collision energy (NCE) 32%. The isolation window was 1.2 m/z and first mass was fixed at 100 m/z.

Data Interpretation and protein identification

The annotated *Clostridium autoethanogenum* DSM 10061 database (Humphreys *et al.*, 2015), (Taxonomy ID 1341692) was downloaded from National Center for Biotechnology Information (NCBI) repository (accession number CP012395), and uploaded on MaxQuant software (version 1.5.2.8) with the following settings; For “type the experimental set”, the reporter ion MS2 including 4 plex iTRAQ were selected with reporter mass tolerant 0.1 Da. Specifications including digestion by trypsin and two miss cleavage was allowed per peptide. In variable modifications, oxidation of methionine and deamidation (NQ) of asparagine and glutamine were selected. In a fixed modification, methylthio of cysteine was set. The false discovery rate (FDR) at the peptide spectrum match/protein level was set at 1%. The reporter ions intensities (114, 115, 116, and 117) were then exported to Excel. iTRAQ ratios used for relative quantification was determined by applying an in-house data analysis pipeline as previously described (Ow *et al.*, 2009) and protein quantification values were obtained in log space. Isotopic and median corrections were applied to the reporter intensity ions to compensate systematic errors between the labels and to determine fold changes of differentially abundant proteins at *p*-value 0.05, as a statistical method described by Pham et al. (Pham *et al.*, 2010).

Enzyme assays

Specific activity measurements

Protocols followed previously adapted methods (Wang *et al.*, 2013; Mock *et al.*, 2015). Enzyme activities were performed at 37 °C, with the use of 3.5 mL OS glass cuvettes (Hellma Analytics, Germany), measured using a Specord 250 spectrophotometer (AnalytikJena AG, Germany). Cuvettes were sealed with silicone stoppers to ensure gas impermeability, while allowing needles to pass through. Buffers prepared for each assay were done in a strictly anaerobic environment using sealed 100 mL serum bottles. Stock solutions of 1 M methyl viologen, 1 M DTT and 1 M sodium formate were mixed using anaerobic distilled water, and sparged with N2. Buffers for each assay were made with 100 mM Tris-HCl (pH7.5) using anaerobic dH_2_O in 100 mL sealed serum bottles. On the day of the experiment 10 mM methyl viologen and 2mM DTT was added. Separate bottles of buffers for each assay were made and dependant on the assay were sparged with either pure CO, H_2_ or N_2_ gas.

CO dehydrogenase assay

Cuvette assay mixture contained 100 mM Tris-HCl, 10 mM methyl viologen and 2 mM DTT. Cuvettes were sparged with CO for 5 min to ensure a 100% CO gas phase. Cuvettes were placed into the spectrophotometer and the background was measured for up to 60 sec, subsequently varying amounts of crude enzyme extract were injected into the cuvettes and measured immediately. As a negative control, a set of cuvettes containing N_2_-sparged buffer, as well as 100% N_2_ gas-phase were used.

Hydrogenase assay

Cuvette assay mixture contained 100 mM Tris-HCl, 10 mM methyl viologen and 2 mM DTT. Cuvettes were sparged with H_2_ for 5 min to ensure a 100% H_2_ gas phase. Cuvettes were placed into the spectrophotometer and the background was measured for up to 60 sec. Varying amounts of crude enzyme extract were injected into the cuvettes and measured immediately. As a negative control, a set of cuvettes containing N_2_-sparged buffer as well as 100% N_2_ gas-phase were used.

Alcohol dehydrogenase

Cuvette assay mixture contained 100 mM potassium phosphate (pH 6), 1.1 mM acetaldehyde, 1 mM NADH and 2 mM DTT. Cuvettes were sparged with N_2_ for 5 min to ensure a 100% N_2_ gas phase. Varying amounts of crude enzyme extract were injected into the cuvettes and measured immediately. As a negative control, cuvettes containing no acetaldehyde were used.

Calculating specific activity

After the start of each reaction, the reduction of methyl viologen with extinction coefficient, ε = 9.8 mM^-1^ cm^-1^ was monitored spectrophotometrically at 578 nm. NADH with extinction coefficient ε = 6.22 mM^-1^cm^-1^ was monitored spectrophotometrically at 340 nm. For the calculation of specific enzyme activity used equation 2.1:

$U= \frac{V\frac{\Delta A}{\min}}{v\varepsilon d}$ (eq. 2.1)

Where U is specific enzyme activity, V is total assay volume, v is volume of crude extract added, $\left( \frac{\Delta A}{min} \right)$ is absorbance change per minutes (the slope), d is the path length (1 cm) and ε is the extinction coefficient. Units are first given as μmol min^−1^ml^−1^, then to obtain a μmol min^−1^mg^−1^ unit (Specific enzyme activity (U)), values were divided by protein concentration.

Negative control samples were deducted from averaged experimental samples for final values. Values were normalised against WT and Δ*agrD1D2* protein concentrations which were determined by Bradford Assay, (Sigma Aldrich, UK).

**Section 3: Full list of proteomic analysis**

Table S6:

| Relative abundance of protein expressions (iTRAQ ratio representing fold changes) between WT and ΔagrD1D2. Negative values indicate reduced abundance of proteins and positive values indicates increased abundance of proteins. | | | | |
| --- | --- | --- | --- | --- |
| Uniprot ID | **Locus Tag** | **Protein** | ***P* value** | **Fold change** |
| U5RRQ8 | CLAU_0016 | Transcriptional regulator, MarR family | 3.78E-07 | -1.39 |
| U5RNS7 | CLAU_0024 | Rubredoxin domain containing protein | 1.59E-02 | -1.37 |
| U5RNF1 | CLAU_0027 | NAD(+) diphosphatase (EC 3.6.1.22) | 1.46E-03 | 1.19 |
| U5RSB8 | CLAU_0035 | Putative transcriptional regulator, GntR family (EC 2.6.1.57) | 9.89E-03 | 1.18 |
| U5RNW7 | CLAU_0054 | Chaperone protein HtpG (Heat shock protein HtpG) (High temperature protein G) | 1.43E-02 | -1.17 |
| U5RP02 | CLAU_0089 | Aldehyde ferredoxin oxidoreductase (EC 1.2.7.5) | 2.58E-07 | -1.24 |
| U5RP39 | CLAU_0089 | Aldehyde ferredoxin oxidoreductase (EC 1.2.7.5) | 8.78E-05 | -1.73 |
| U5RP39 | CLAU_0099 | Aldehyde ferredoxin oxidoreductase | 3.33E-19 | -2.01 |
| U5RNZ7 | CLAU_0101 | UBA/THIF-type NAD/FAD binding protein | 2.06E-02 | -1.52 |
| U5RP61 | CLAU_0117 | Ketol-acid reductoisomerase (NADP(+)) (KARI) (EC 1.1.1.86) (Acetohydroxy-acid isomeroreductase) (AHIR) (Alpha-keto-beta-hydroxylacyl reductoisomerase) | 9.84E-08 | 1.54 |
| U5RSQ7 | CLAU_0118 | Dihydroxy-acid dehydratase (DAD) (EC 4.2.1.9) | 5.07E-04 | 1.15 |
| U5RST6 | CLAU_0140 | Dihydropteroate synthase DHPS | 1.19E-02 | -1.21 |
| U5RS85 | CLAU_0145 | Cobalamin B12-binding domain protein | 1.04E-02 | -1.3 |
| U5RNV1 | CLAU_0205 | Putative transcriptional regulator, GntR family (EC 2.6.1.57) | 2.31E-04 | 1.34 |
| U5RSY0 | CLAU_0207 | Aminotransferase (EC 2.6.1.-) | 6.88E-06 | 1.17 |
| U5RSE1 | CLAU_0213 | Transcriptional regulator, RpiR family | 4.55E-03 | -1.16 |
| U5RP20 | CLAU_0224 | Histidine ammonia-lyase (Histidase) (EC 4.3.1.3) | 6.41E-04 | -1.37 |
| U5RPF8 | CLAU_0226 | Urocanate hydratase (Urocanase) (EC 4.2.1.49) (Imidazolonepropionate hydrolase) | 6.32E-05 | -1.36 |
| U5RP25 | CLAU_0228 | Glutamate formiminotransferase (EC 2.1.2.5) | 2.74E-03 | 1.31 |
| U5RP41 | CLAU_0304 | Molybdenum ABC transporter, periplasmic molybdate-binding protein | 1.07E-02 | 1.12 |
| U5RP91 | CLAU_0308 | Phosphoribosylaminoimidazolecarboxamide formyltransferase (EC 2.1.2.3) | 8.11E-03 | -1.15 |
| U5RSS2 | CLAU_0370 | (R,R)-butanediol dehydrogenase (EC 1.1.1.4) | 2.67E-34 | 1.57 |
| U5RSS7 | CLAU_0375 | Efflux transporter, RND family, MFP subunit | 1.19E-02 | 1.19 |
| U5RPW4 | CLAU_0385 | Cysteine desulfurase IscS (EC 2.8.1.7) | 3.23E-02 | -1.2 |
| U5RPH4 | CLAU_0388 | Acetolactate synthase (EC 2.2.1.6) | 8.64E-13 | 1.69 |
| U5RPD9 | CLAU_0399 | Nitrogenase iron protein (EC 1.18.6.1) (Nitrogenase Fe protein) (Nitrogenase component II) (Nitrogenase reductase) | 8.29E-03 | 1.43 |
| U5RPK3 | CLAU_0458 | Glutamate synthase (NADPH) (EC 1.4.1.13) | 1.08E-13 | 1.88 |
| U5RPK9 | CLAU_0463 | NADPH dehydrogenase (EC 1.6.99.1) | 8.52E-05 | 1.21 |
| U5RTL9 | CLAU_0465 | Cyclase family protein | 1.02E-09 | 1.75 |
| U5RQB1 | CLAU_0532 | Alcohol dehydrogenase zinc-binding domain protein | 1.44E-02 | 1.3 |
| U5RTC4 | CLAU_0568 | Carbamoyl-phosphate synthase small chain (EC 6.3.5.5) (Carbamoyl-phosphate synthetase glutamine chain) | 1.52E-02 | 1.46 |
| U5RPZ5 | CLAU_0569 | Ornithine carbamoyltransferase (OTCase) (EC 2.1.3.3) | 1.99E-08 | 1.54 |
| U5RQX8 | CLAU_0711 | Glycerol dehydrogenase (EC 1.1.1.6) | 6.99E-20 | 2.16 |
| U5RQH8 | CLAU_0725 | Uncharacterized protein | 2.36E-02 | 1.36 |
| U5RTY0 | CLAU_0729 | Glutamine--tRNA ligase (EC 6.1.1.18) (Glutaminyl-tRNA synthetase) (GlnRS) | 2.10E-06 | 1.24 |
| U5RUF8 | CLAU_0733 | DegT/DnrJ/EryC1/StrS aminotransferase | 1.50E-05 | -1.37 |
| U5RTY5 | CLAU_0734 | Glutamine--scyllo-inositol transaminase (EC 2.6.1.50) | 2.81E-02 | -1.16 |
| U5RR17 | CLAU_0752 | Uncharacterized protein | 1.23E-07 | 1.55 |
| U5RU29 | CLAU_0789 | Glucarate dehydratase (EC 4.2.1.40) | 3.06E-09 | -1.42 |
| U5RUL7 | CLAU_0793 | Methylglyoxal reductase (NADPH-dependent) (EC 1.1.1.283) | 3.97E-11 | -1.75 |
| U5RQN3 | CLAU_0795 | Dihydrodipicolinate synthase (EC 4.3.3.7) | 6.07E-09 | -1.57 |
| U5RUT7 | CLAU_0865 | Cardiolipin synthase (CL synthase) (EC 2.7.8.-) | 1.08E-03 | 1.32 |
| U5RQT1 | CLAU_0873 | Shikimate dehydrogenase (NADP(+)) (SDH) (EC 1.1.1.25) | 4.60E-03 | 1.21 |
| U5RRE0 | CLAU_0879 | Phospho-2-dehydro-3-deoxyheptonate aldolase (EC 2.5.1.54) | 4.04E-08 | -1.21 |
| U5RUV6 | CLAU_0886 | UPF0210 protein CAETHG_0918 | 1.11E-06 | 1.21 |
| U5RUG4 | CLAU_0940 | NADPH-dependent FMN reductase | 3.06E-04 | -1.91 |
| U5RUQ1 | CLAU_1022 | NADP-dependent oxidoreductase domain containing protein | 1.45E-02 | 1.52 |
| U5RRT3 | CLAU_1024 | Putative methyl-accepting chemotaxis sensory transducer | 2.48E-02 | 1.2 |
| U5RRK2 | CLAU_1071 | Cob(I)alamin adenosyltransferase | 1.56E-02 | -1.16 |
| U5RS07 | CLAU_1078 | Precorrin-2 C20-methyltransferase | 1.27E-04 | -1.23 |
| U5RS12 | CLAU_1083 | Nicotinate-nucleotide--dimethylbenzimidazole phosphoribosyltransferase (NN:DBI PRT) (EC 2.4.2.21) (N(1)-alpha-phosphoribosyltransferase) | 1.12E-04 | -1.23 |
| U5RVJ6 | CLAU_1084 | Cobyrinate a,c-diamide synthase (EC 6.3.5.11) (Cobyrinic acid a,c-diamide synthetase) | 8.04E-03 | -1.21 |
| U5RVL0 | CLAU_1099 | Lipoprotein | 1.26E-05 | 1.12 |
| U5RRK7 | CLAU_1107 | Nitroreductase | 2.68E-04 | 1.24 |
| U5RS69 | CLAU_1154 | ATP-dependent Clp protease proteolytic subunit (EC 3.4.21.92) (Endopeptidase Clp) | 1.81E-02 | 1.15 |
| U5RS79 | CLAU_1164 | Phosphomethylpyrimidine kinase (EC 2.7.4.7) | 2.31E-02 | 1.77 |
| U5RRR0 | CLAU_1173 | ABC-type transporter, periplasmic subunit family 3 | 2.32E-08 | 1.23 |
| U5RS92 | CLAU_1179 | Threonine synthase (EC 4.2.3.1) | 1.78E-02 | 1.07 |
| U5RVS7 | CLAU_1185 | Cold-shock DNA-binding domain protein | 1.99E-09 | -1.25 |
| U5RVD2 | CLAU_1226 | Aspartyl-tRNA synthetase (EC 6.1.1.12) | 1.30E-05 | -1.17 |
| U5RS67 | CLAU_1305 | 50S ribosomal protein L35 | 5.95E-03 | 1.48 |
| U5RSK7 | CLAU_1307 | Threonine--tRNA ligase (EC 6.1.1.3) (Threonyl-tRNA synthetase) (ThrRS) | 8.77E-09 | -1.29 |
| U5RW61 | CLAU_1332 | Phosphoglycerate dehydrogenase (EC 1.1.1.95) | 5.72E-09 | 2.64 |
| U5RVM6 | CLAU_1333 | Dimethylmenaquinone methyltransferase | 8.86E-12 | 2.68 |
| U5RS58 | CLAU_1335 | Amidohydrolase | 4.02E-06 | 1.8 |
| U5RVQ0 | CLAU_1358 | CRISPR-associated protein | 3.39E-03 | 1.37 |
| U5RWD0 | CLAU_1413 | Transcription elongation factor GreA (Transcript cleavage factor GreA) | 4.15E-03 | -1.2 |
| U5RSV4 | CLAU_1417 | Uncharacterized protein | 4.89E-08 | 1.42 |
| U5RSW5 | CLAU_1432 | ATP-dependent Clp protease proteolytic subunit (EC 3.4.21.92) (Endopeptidase Clp) | 4.34E-04 | 1.19 |
| U5RSK1 | CLAU_1435 | Uncharacterized protein | 8.05E-03 | 1.1 |
| U5RSG1 | CLAU_1436 | Orotate phosphoribosyltransferase (OPRT) (OPRTase) (EC 2.4.2.10) | 3.15E-04 | -1.28 |
| U5RSK5 | CLAU_1440 | Aspartate carbamoyltransferase regulatory subunit | 1.07E-03 | -1.53 |
| U5RSG6 | CLAU_1441 | Aspartate carbamoyltransferase (EC 2.1.3.2) (Aspartate transcarbamylase) (ATCase) | 2.55E-06 | -1.41 |
| U5RSK0 | CLAU_1477 | Hemerythrin-like metal-binding protein | 1.01E-02 | -1.56 |
| U5RWQ1 | CLAU_1529 | GMP synthase [glutamine-hydrolyzing] (EC 6.3.5.2) (GMP synthetase) (Glutamine amidotransferase) | 4.42E-04 | -1.13 |
| U5RW54 | CLAU_1530 | Inosine-5'-monophosphate dehydrogenase (IMP dehydrogenase) (IMPD) (IMPDH) (EC 1.1.1.205) | 1.95E-13 | -1.16 |
| U5RSU4 | CLAU_1531 | 60 kDa chaperonin (GroEL protein) (Protein Cpn60) | 1.41E-10 | -1.13 |
| U5RW58 | CLAU_1535 | Hydrogenase, Fe-only (EC 1.12.7.2) | 6.67E-04 | -1.36 |
| U5RSQ6 | CLAU_1537 | NADH dehydrogenase (Ubiquinone) 24 kDa subunit | 8.08E-05 | -1.64 |
| U5RW67 | CLAU_1545 | 3-hydroxybutyryl-CoA dehydrogenase (EC 1.1.1.157) | 1.45E-02 | -1.36 |
| U5RT85 | CLAU_1553 | Pyruvate carboxylase (EC 6.4.1.1) | 8.04E-07 | 1.24 |
| U5RW82 | CLAU_1561 | Uncharacterized protein | 6.65E-03 | -1.41 |
| U5RWW1 | CLAU_1565 | Glycine cleavage system H protein | 1.23E-03 | -1.18 |
| U5RWA4 | CLAU_1566 | CO dehydrogenase/acetyl-CoA synthase complex, beta subunit (EC 2.3.1.169) | 2.48E-47 | -1.4 |
| U5RWC1 | CLAU_1571 | Dihydrolipoyl dehydrogenase (EC 1.8.1.4) | 1.02E-07 | -1.16 |
| U5RT18 | CLAU_1572 | Methylenetetrahydrofolate reductase (EC 1.5.1.20) | 2.57E-04 | 1.11 |
| U5RWC6 | CLAU_1576 | Formate--tetrahydrofolate ligase (EC 6.3.4.3) (Formyltetrahydrofolate synthetase) (FHS) (FTHFS) | 1.09E-07 | -1.08 |
| U5RSY1 | CLAU_1578 | Carbon-monoxide dehydrogenase (Acceptor) (EC 1.2.99.2) | 1.04E-13 | -2.96 |
| U5RTE2 | CLAU_1579 | Carbon-monoxide dehydrogenase (Acceptor) (EC 1.2.99.2) | 1.34E-30 | -2.26 |
| U5RT31 | CLAU_1587 | Nitrite and sulfite reductase 4Fe-4S region | 4.68E-04 | 1.4 |
| U5RT38 | CLAU_1647 | Aspartokinase (EC 2.7.2.4) | 1.53E-02 | 1.09 |
| U5RTD3 | CLAU_1710 | Enolase (EC 4.2.1.11) (2-phospho-D-glycerate hydro-lyase) (2-phosphoglycerate dehydratase) | 6.94E-06 | 1.09 |
| U5RTA1 | CLAU_1711 | 2,3-bisphosphoglycerate-independent phosphoglycerate mutase (BPG-independent PGAM) (Phosphoglyceromutase) (iPGM) (EC 5.4.2.12) | 2.52E-04 | 1.1 |
| U5RTQ5 | CLAU_1712 | Triosephosphate isomerase (TIM) (TPI) (EC 5.3.1.1) (Triose-phosphate isomerase) | 1.02E-04 | 1.11 |
| U5RX94 | CLAU_1713 | Phosphoglycerate kinase (EC 2.7.2.3) | 1.19E-04 | 1.09 |
| U5RWQ9 | CLAU_1714 | Glyceraldehyde-3-phosphate dehydrogenase (EC 1.2.1.-) | 7.44E-18 | 1.14 |
| U5RTE9 | CLAU_1729 | Cysteine synthase (EC 2.5.1.47) | 4.20E-09 | 2 |
| U5RTS5 | CLAU_1731 | Ig domain protein | 2.42E-02 | -1.24 |
| U5RXB0 | CLAU_1732 | Rubrerythrin | 1.04E-10 | -1.65 |
| U5RXD6 | CLAU_1757 | Pyridoxal 5'-phosphate synthase subunit PdxS (PLP synthase subunit PdxS) (EC 4.3.3.6) (Pdx1) | 3.36E-10 | 1.19 |
| U5RWU8 | CLAU_1758 | Pyridoxal 5'-phosphate synthase subunit PdxT (EC 4.3.3.6) (Pdx2) (Pyridoxal 5'-phosphate synthase glutaminase subunit) (EC 3.5.1.2) | 5.81E-04 | 1.38 |
| U5RTV6 | CLAU_1766 | Alcohol dehydrogenase (EC 1.1.1.1) | 8.71E-21 | 2.59 |
| U5RTI0 | CLAU_1769 | Microcompartments protein | 1.41E-15 | 3.77 |
| U5RTI0 | CLAU_1769 | Microcompartments protein | 2.64E-16 | 2.59 |
| U5RTW1 | CLAU_1771 | Phosphate propanoyltransferase (EC 2.3.1.222) | 3.85E-05 | 3.36 |
| U5RXL2 | CLAU_1771 | Phosphate propanoyltransferase (EC 2.3.1.222) | 5.29E-09 | 2.53 |
| U5RTF0 | CLAU_1772 | Microcompartments protein | 3.31E-16 | 2.93 |
| U5RXF4 | CLAU_1772 | Acetaldehyde dehydrogenase (Acetylating) | 7.36E-04 | 1.96 |
| U5RWV7 | CLAU_1773 | Microcompartments protein | 1.57E-05 | 3.22 |
| U5RTI4 | CLAU_1774 | Ethanolamine utilization EutQ family protein | 6.79E-07 | 2.63 |
| U5RTF4 | CLAU_1775 | Microcompartments protein | 1.83E-09 | 2.18 |
| U5RTW4 | CLAU_1776 | Respiratory-chain NADH dehydrogenase domain 51 kDa subunit | 9.75E-16 | 2.88 |
| U5RXP0 | CLAU_1779 | Ethanolamine utilization protein EutJ family protein | 3.09E-06 | 2.25 |
| U5RTF7 | CLAU_1780 | Uncharacterized protein | 1.67E-02 | 2.46 |
| U5RTW9 | CLAU_1781 | Choline trimethylamine-lyase activating enzyme (EC 1.97.1.-) (Choline utilization protein D) (GRE activase CutD) (Glycyl-radical enzyme activating enzyme CutD) (GRE activating enzyme CutD) | 4.78E-03 | 1.67 |
| U5RXG3 | CLAU_1782 | Choline trimethylamine-lyase (Choline TMA-lyase) (EC 4.3.99.4) (Choline utilization protein C) | 1.29E-43 | 2.86 |
| U5RWW6 | CLAU_1783 | Acetaldehyde dehydrogenase (Acetylating) (EC 1.2.1.10) | 1.80E-13 | 2.48 |
| U5RTJ3 | CLAU_1784 | Microcompartments protein | 1.14E-08 | 2.56 |
| U5RTG1 | CLAU_1785 | Microcompartments protein | 1.17E-08 | 2.13 |
| U5RTX4 | CLAU_1786 | Uncharacterized protein | 3.46E-05 | 3.93 |
| U5RWX1 | CLAU_1788 | Uncharacterized protein | 3.21E-05 | 2.41 |
| U5RTK2 | CLAU_1794 | Alcohol dehydrogenase (EC 1.1.1.1) | 1.59E-10 | -2.13 |
| U5RU23 | CLAU_1845 | Thioredoxin | 7.96E-03 | -1.13 |
| U5RTQ9 | CLAU_1848 | Phosphoenolpyruvate-protein phosphotransferase (EC 2.7.3.9) (Phosphotransferase system, enzyme I) | 2.18E-04 | 1.72 |
| U5RXN5 | CLAU_1856 | Methylaspartate ammonia-lyase (EC 4.3.1.2) | 1.13E-05 | -1.18 |
| U5RX36 | CLAU_1857 | Glutamate mutase epsilon subunit (EC 5.4.99.1) (Glutamate mutase E chain) (Glutamate mutase large subunit) (Methylaspartate mutase) | 2.06E-03 | -1.13 |
| U5RU43 | CLAU_1865 | 30S ribosomal protein S9 | 4.54E-04 | -1.09 |
| U5RXP1 | CLAU_1866 | 50S ribosomal protein L13 | 3.98E-07 | -1.21 |
| U5RXP5 | CLAU_1871 | 50S ribosomal protein L17 | 9.09E-03 | -1.14 |
| U5RX48 | CLAU_1872 | DNA-directed RNA polymerase subunit alpha (RNAP subunit alpha) (EC 2.7.7.6) (RNA polymerase subunit alpha) (Transcriptase subunit alpha) | 1.68E-04 | -1.1 |
| U5RTT2 | CLAU_1873 | 30S ribosomal protein S4 | 1.52E-02 | -1.11 |
| U5RU54 | CLAU_1875 | 30S ribosomal protein S13 | 2.71E-03 | -1.16 |
| U5RTT7 | CLAU_1878 | Adenylate kinase (AK) (EC 2.7.4.3) (ATP-AMP transphosphorylase) (ATP:AMP phosphotransferase) (Adenylate monophosphate kinase) | 2.31E-03 | -1.15 |
| U5RU58 | CLAU_1880 | 50S ribosomal protein L15 | 4.72E-05 | -1.27 |
| U5RX57 | CLAU_1882 | 30S ribosomal protein S5 | 3.82E-04 | -1.15 |
| U5RTR1 | CLAU_1884 | 50S ribosomal protein L6 | 1.39E-08 | -1.24 |
| U5RX62 | CLAU_1887 | 50S ribosomal protein L5 | 6.20E-05 | -1.13 |
| U5RTU8 | CLAU_1888 | 50S ribosomal protein L24 | 7.27E-03 | -1.2 |
| U5RTR7 | CLAU_1889 | 50S ribosomal protein L14 | 9.37E-03 | -1.22 |
| U5RU69 | CLAU_1890 | 30S ribosomal protein S17 | 6.46E-03 | -1.27 |
| U5RU73 | CLAU_1895 | 30S ribosomal protein S19 | 5.34E-05 | -1.17 |
| U5RX69 | CLAU_1897 | 50S ribosomal protein L23 | 1.53E-02 | -1.14 |
| U5RU77 | CLAU_1900 | 30S ribosomal protein S10 | 2.92E-04 | -1.18 |
| U5RU93 | CLAU_1901 | Elongation factor Tu (EF-Tu) | 3.75E-03 | -1.09 |
| U5RX73 | CLAU_1902 | Elongation factor G (EF-G) | 4.16E-14 | -1.24 |
| U5RTW7 | CLAU_1908 | 50S ribosomal protein L7/L12 | 1.44E-10 | -1.23 |
| U5RTT9 | CLAU_1909 | 50S ribosomal protein L10 | 8.37E-07 | -1.27 |
| U5RU88 | CLAU_1910 | 50S ribosomal protein L1 | 1.55E-03 | -1.2 |
| U5RXU4 | CLAU_1926 | ATPase AAA-2 domain protein | 3.42E-14 | -1.34 |
| U5RX93 | CLAU_1927 | Protein-arginine kinase (EC 2.7.14.1) | 2.97E-04 | -1.58 |
| U5RTY8 | CLAU_1928 | UvrB/UvrC protein | 7.07E-07 | -1.52 |
| U5RX97 | CLAU_1932 | UDP-N-acetylmuramoylalanine--D-glutamate ligase (EC 6.3.2.9) (D-glutamic acid-adding enzyme) (UDP-N-acetylmuramoyl-L-alanyl-D-glutamate synthetase) | 2.32E-02 | 1.1 |
| U5RTZ3 | CLAU_1933 | Glycine--tRNA ligase (EC 6.1.1.14) (Glycyl-tRNA synthetase) (GlyRS) | 3.72E-05 | 1.26 |
| U5RTW6 | CLAU_1934 | Lysine--tRNA ligase (EC 6.1.1.6) (Lysyl-tRNA synthetase) (LysRS) | 8.06E-03 | -1.09 |
| U5RUB0 | CLAU_1935 | Transcription elongation factor GreA (Transcript cleavage factor GreA) | 1.66E-02 | -1.13 |
| U5RUB5 | CLAU_1940 | Hypoxanthine phosphoribosyltransferase (EC 2.4.2.8) | 2.35E-02 | 1.3 |
| U5RU02 | CLAU_1943 | RNA binding S1 domain protein | 1.23E-02 | -1.23 |
| U5RXB8 | CLAU_1952 | Foldase protein PrsA (EC 5.2.1.8) | 2.93E-04 | 1.13 |
| U5RU17 | CLAU_1958 | Ribose-phosphate pyrophosphokinase (RPPK) (EC 2.7.6.1) (5-phospho-D-ribosyl alpha-1-diphosphate) (Phosphoribosyl diphosphate synthase) (Phosphoribosyl pyrophosphate synthase) (P-Rib-PP synthase) (PRPP synthase) (PRPPase) | 1.52E-07 | -1.29 |
| U5RU03 | CLAU_1974 | Helix-turn-helix domain protein | 1.65E-02 | -1.5 |
| U5RY03 | CLAU_1976 | Glutamine synthetase catalytic region | 1.06E-14 | 1.52 |
| U5RU44 | CLAU_1982 | SEC-C motif domain protein | 1.28E-03 | -1.25 |
| U5RY25 | CLAU_1995 | Acetyl-CoA carboxylase, biotin carboxyl carrier protein | 2.01E-04 | -1.23 |
| U5RY43 | CLAU_2009 | Adenylosuccinate synthetase (AMPSase) (AdSS) (EC 6.3.4.4) (IMP--aspartate ligase) | 1.08E-02 | -1.1 |
| U5RUI7 | CLAU_2018 | Cupin 2 conserved barrel domain protein | 1.18E-05 | 1.43 |
| U5RY61 | CLAU_2024 | Putative agmatine deiminase (EC 3.5.3.12) (Agmatine iminohydrolase) | 1.80E-04 | -2.05 |
| U5RU95 | CLAU_2030 | Carbamate kinase | 2.53E-02 | -2.15 |
| U5RU62 | CLAU_2031 | Putrescine carbamoyltransferase (PTC) (PTCase) (EC 2.1.3.6) (Putrescine transcarbamoylase) (Putrescine transcarbamylase) | 2.02E-03 | -2.02 |
| U5RY88 | CLAU_2051 | 30S ribosomal protein S18 | 1.20E-04 | -1.38 |
| U5RUB3 | CLAU_2053 | 30S ribosomal protein S6 | 1.13E-02 | -1.15 |
| U5RU85 | CLAU_2054 | Uncharacterized protein | 1.43E-02 | 1.34 |
| U5RYF0 | CLAU_2081 | UPF0182 protein CAETHG_2133 | 8.62E-04 | 1.17 |
| U5RYK0 | CLAU_2147 | Nucleoid-associated protein CAETHG_2200 | 2.00E-03 | 1.26 |
| U5RYK5 | CLAU_2157 | Alanine--glyoxylate transaminase (EC 2.6.1.44) | 2.03E-04 | -1.22 |
| U5RY08 | CLAU_2158 | Phosphoglycerate dehydrogenase (EC 1.1.1.95) | 1.06E-11 | -2.8 |
| U5RV15 | CLAU_2172 | Peptidylprolyl isomerase (EC 5.2.1.8) | 9.60E-04 | -1.26 |
| U5RYN7 | CLAU_2202 | Transcriptional regulator, AbrB family | 3.40E-05 | -1.24 |
| U5RUS5 | CLAU_2204 | Uncharacterized protein | 1.49E-02 | 1.51 |
| U5RUP7 | CLAU_2205 | Phage shock protein A, PspA | 2.76E-14 | 1.3 |
| U5RUQ8 | CLAU_2220 | Methionine--tRNA ligase (EC 6.1.1.10) (Methionyl-tRNA synthetase) (MetRS) | 7.56E-03 | -1.15 |
| U5RYR3 | CLAU_2231 | Anaerobic ribonucleoside-triphosphate reductase (EC 1.17.4.2) | 1.75E-02 | -1.2 |
| U5RUU9 | CLAU_2267 | CTP synthase (EC 6.3.4.2) (Cytidine 5'-triphosphate synthase) (Cytidine triphosphate synthetase) (CTP synthetase) (CTPS) (UTP--ammonia ligase) | 5.04E-03 | -1.16 |
| U5RYB0 | CLAU_2270 | 50S ribosomal protein L31 | 8.30E-03 | -1.21 |
| U5RYX1 | CLAU_2289 | ATP synthase subunit alpha (EC 3.6.3.14) (ATP synthase F1 sector subunit alpha) (F-ATPase subunit alpha) | 1.36E-06 | 1.15 |
| U5RV03 | CLAU_2291 | ATP synthase subunit beta (EC 3.6.3.14) (ATP synthase F1 sector subunit beta) (F-ATPase subunit beta) | 2.52E-03 | 1.07 |
| U5RYD5 | CLAU_2300 | S-adenosylmethionine synthase (AdoMet synthase) (EC 2.5.1.6) (MAT) (Methionine adenosyltransferase) | 2.74E-07 | 1.43 |
| U5RV19 | CLAU_2305 | Protein translocase subunit SecA | 1.65E-03 | -1.15 |
| U5RYZ2 | CLAU_2308 | Glutamate dehydrogenase | 1.21E-07 | -1.27 |
| U5RZ09 | CLAU_2322 | Fructose-1,6-bisphosphate aldolase, class II (EC 4.1.2.13) | 2.33E-05 | -1.14 |
| U5RZ41 | CLAU_2360 | Cell division ATP-binding protein FtsE (EC 3.6.3.28) | 2.81E-02 | 1.24 |
| U5RVL5 | CLAU_2378 | Pyruvate kinase (EC 2.7.1.40) | 3.27E-16 | 1.26 |
| U5RZ70 | CLAU_2394 | Methyl-accepting chemotaxis sensory transducer | 1.09E-03 | 1.15 |
| U5RVP0 | CLAU_2404 | Nitrogen regulatory protein P-II | 8.36E-04 | -1.47 |
| U5RYS5 | CLAU_2445 | Carbamoyl-phosphate synthase large chain (EC 6.3.5.5) (Carbamoyl-phosphate synthetase ammonia chain) | 2.17E-02 | 1.16 |
| U5RVG8 | CLAU_2492 | Cell wall binding repeat 2-containing protein | 8.50E-113 | 1.36 |
| U5RVI8 | CLAU_2512 | Cell wall binding repeat 2-containing protein | 2.27E-03 | 1.58 |
| U5RZK5 | CLAU_2519 | Outer membrane efflux protein | 7.94E-03 | 1.18 |
| U5RZ08 | CLAU_2520 | ResB family protein | 1.11E-02 | 1.22 |
| U5RW09 | CLAU_2523 | Cell wall binding repeat 2-containing protein | 5.52E-49 | 1.48 |
| U5RVZ8 | CLAU_2590 | Tubulin-like protein | 3.07E-06 | 1.36 |
| U5RW20 | CLAU_2613 | D-lactate dehydrogenase (Cytochrome) (EC 1.1.2.4) | 1.90E-02 | 1.36 |
| U5RW30 | CLAU_2623 | Gamma-glutamyl phosphate reductase (GPR) (EC 1.2.1.41) (Glutamate-5-semialdehyde dehydrogenase) (Glutamyl-gamma-semialdehyde dehydrogenase) (GSA dehydrogenase) | 1.20E-20 | 1.33 |
| U5RZI2 | CLAU_2641 | Chaperone protein ClpB | 1.57E-04 | 1.17 |
| U5RW10 | CLAU_2653 | Dihydropteroate synthase (DHPS) (EC 2.5.1.15) (Dihydropteroate pyrophosphorylase) | 3.74E-05 | -1.4 |
| U5RW34 | CLAU_2677 | O-acetylhomoserine/O-acetylserine sulfhydrylase (EC 2.5.1.47) | 1.38E-26 | 1.29 |
| U5RZL7 | CLAU_2680 | ABC-type transporter, periplasmic subunit family 3 | 7.95E-06 | 1.16 |
| U5RWL5 | CLAU_2683 | Argininosuccinate synthase | 4.86E-14 | 1.63 |
| U5S045 | CLAU_2683 | Argininosuccinate synthase (EC 6.3.4.5) | 1.01E-02 | 1.27 |
| U5RW42 | CLAU_2686 | Transcriptional regulator, AsnC family | 1.19E-02 | -1.83 |
| U5RW59 | CLAU_2707 | Band 7 protein | 3.77E-03 | 1.2 |
| U5RWP5 | CLAU_2713 | Formate dehydrogenase, alpha subunit (EC 1.7.99.4) | 6.46E-04 | -1.38 |
| S5ZJ59 | CLAU_2717 | Electron bifurcating FeFe-hydrogenase dependent on TPN subunit C (NADH-quinone oxidoreductase, E subunit) (EC 1.6.99.5) | 1.02E-05 | -1.46 |
| S5Z7X0 | CLAU_2719 | 4Fe-4S ferredoxin, iron-sulpur binding domain-containing protein (Electron bifurcating FeFe-hydrogenase dependent on TPN subunit D) | 6.38E-04 | -1.27 |
| U5RWA6 | CLAU_2721 | Hydrogenase, Fe-only (EC 1.12.7.2) | 1.16E-08 | -1.29 |
| U5RW72 | CLAU_2722 | 4Fe-4S ferredoxin, iron-sulpur binding domain-containing protein | 4.02E-03 | -1.3 |
| U5S087 | CLAU_2728 | UPF0735 ACT domain-containing protein CAETHG_2806 | 1.38E-04 | 1.33 |
| U5RW84 | CLAU_2736 | Cell shape determining protein, MreB/Mrl family | 2.91E-03 | 1.16 |
| U5RWD3 | CLAU_2750 | 50S ribosomal protein L21 | 3.62E-05 | -1.39 |
| U5RWT5 | CLAU_2752 | 50S ribosomal protein L27 | 4.63E-03 | -1.12 |
| U5RW98 | CLAU_2756 | Endoribonuclease L-PSP | 5.86E-05 | 1.25 |
| U5RWF3 | CLAU_2764 | Dihydropteroate synthase DHPS | 2.43E-03 | -1.44 |
| U5RWA7 | CLAU_2765 | Uncharacterized protein | 4.84E-03 | -1.92 |
| U5S0E6 | CLAU_2788 | Hydroxylamine reductase (EC 1.7.99.1) (Hybrid-cluster protein) (HCP) (Prismane protein) | 9.27E-09 | 1.23 |
| U5S0G1 | CLAU_2804 | 30S ribosomal protein S20 | 4.89E-03 | -1.11 |
| U5RWJ5 | CLAU_2811 | Protein GrpE (HSP-70 cofactor) | 5.98E-04 | -1.24 |
| U5RZY4 | CLAU_2819 | Uncharacterized protein | 3.51E-03 | -1.13 |
| U5RWJ7 | CLAU_2855 | PDZ/DHR/GLGF domain protein | 6.29E-04 | 1.42 |
| U5RWK6 | CLAU_2865 | Hydrolase | 1.21E-03 | 1.09 |
| U5S0L4 | CLAU_2867 | N5-carboxyaminoimidazole ribonucleotide mutase (N5-CAIR mutase) (EC 5.4.99.18) (5-(carboxyamino)imidazole ribonucleotide mutase) | 1.16E-02 | -1.39 |
| U5RWQ3 | CLAU_2869 | Amidophosphoribosyltransferase (ATase) (EC 2.4.2.14) (Glutamine phosphoribosylpyrophosphate amidotransferase) (GPATase) | 7.41E-03 | -1.17 |
| U5RWS4 | CLAU_2889 | Uncharacterized protein | 4.80E-03 | 1.23 |
| U5S0R8 | CLAU_2924 | Carbon monoxide dehydrogenase (EC 1.2.7.4) | 2.50E-05 | -1.72 |
| U5RXA5 | CLAU_2947 | Pyruvate-flavodoxin oxidoreductase (EC 1.2.7.-) | 1.15E-39 | 1.51 |
| U5RXB1 | CLAU_2952 | CheW protein | 1.32E-02 | -1.17 |
| U5S0V6 | CLAU_2958 | Response regulator receiver protein | 1.49E-02 | 1.16 |
| U5RXI1 | CLAU_3017 | Homoserine dehydrogenase (EC 1.1.1.3) | 9.87E-04 | 1.21 |
| U5RXB4 | CLAU_3055 | Putative signal transduction protein with CBS domain containing protein (EC 3.6.1.1) | 2.55E-03 | 1.14 |
| U5S1B3 | CLAU_3078 | 5'-methylthioadenosine/S-adenosylhomocysteine nucleosidase (EC 3.2.2.9) | 7.86E-03 | 1.36 |
| U5RXJ6 | CLAU_3144 | Electron transport complex subunit C | 9.19E-13 | 1.65 |
| U5RXZ2 | CLAU_3146 | Electron transport complex subunit G | 1.98E-04 | 1.5 |
| U5RXK0 | CLAU_3149 | Electron transport complex, RnfABCDGE type, B subunit | 1.96E-05 | 1.38 |
| U5S1J7 | CLAU_3187 | Uncharacterized protein | 3.41E-05 | 1.38 |
| U5RXN3 | CLAU_3189 | Histidinol phosphate phosphatase HisJ family (EC 3.1.3.15) | 1.13E-02 | 1.2 |
| U5S1K9 | CLAU_3202 | Ethanolamine utilization EutQ family protein | 1.57E-05 | 2.42 |
| U5RXF4 | CLAU_3204 | Acetaldehyde dehydrogenase (Acetylating) (EC 1.2.1.10) | 8.98E-14 | 2.51 |
| U5RXL6 | CLAU_3210 | Cysteine desulfurase IscS (EC 2.8.1.7) | 1.67E-03 | -1.2 |
| U5RY52 | CLAU_3211 | FeS cluster assembly scaffold protein NifU | 1.28E-03 | -1.23 |
| U5RXL9 | CLAU_3215 | UPF0297 protein CAETHG_3298 | 7.22E-05 | -2.04 |
| U5RY95 | CLAU_3256 | Methionyl-tRNA formyltransferase (EC 2.1.2.9) | 6.44E-03 | 1.39 |
| U5RXP7 | CLAU_3260 | Protein serine/threonine phosphatase | 9.09E-04 | 1.31 |
| U5RXQ1 | CLAU_3265 | 50S ribosomal protein L28 | 9.73E-03 | -1.17 |
| U5RXR0 | CLAU_3274 | Phosphate acetyltransferase (EC 2.3.1.8) | 3.01E-03 | -1.09 |
| U5RYD2 | CLAU_3300 | GTP-sensing transcriptional pleiotropic repressor CodY | 6.07E-03 | -1.17 |
| U5S1B8 | CLAU_3302 | Elongation factor Ts (EF-Ts) | 2.14E-04 | -1.12 |
| U5S1W2 | CLAU_3311 | Transcription termination/antitermination protein NusA | 1.16E-02 | -1.14 |
| U5RXV2 | CLAU_3319 | 30S ribosomal protein S15 | 6.54E-04 | -1.21 |
| U5RYE8 | CLAU_3320 | Polyribonucleotide nucleotidyltransferase (EC 2.7.7.8) (Polynucleotide phosphorylase) (PNPase) | 8.05E-07 | -1.24 |
| U5RY13 | CLAU_3333 | Aminotransferase (EC 2.6.1.-) | 1.09E-03 | -1.11 |
| U5RXW4 | CLAU_3334 | Phosphotransferase system, phosphocarrier protein HPr | 4.90E-08 | 1.5 |
| U5RYK8 | CLAU_3384 | Methyl-accepting chemotaxis sensory transducer | 3.75E-05 | -1.25 |
| U5RYA1 | CLAU_3416 | Cupin 2 conserved barrel domain protein | 6.61E-03 | 1.28 |
| U5S1Y5 | CLAU_3521 | Threonine dehydratase | 1.08E-08 | 1.51 |
| U5RYX9 | CLAU_3612 | Glutamine amidotransferase of anthranilate synthase (EC 4.1.3.27) | 8.98E-04 | -1.23 |
| U5RYS2 | CLAU_3613 | Anthranilate phosphoribosyltransferase (EC 2.4.2.18) | 1.98E-04 | -1.25 |
| U5RZB6 | CLAU_3614 | Indole-3-glycerol phosphate synthase (IGPS) (EC 4.1.1.48) | 6.99E-04 | -1.37 |
| U5S2S9 | CLAU_3615 | N-(5'-phosphoribosyl)anthranilate isomerase (PRAI) (EC 5.3.1.24) | 5.10E-03 | -1.26 |
| U5S2B5 | CLAU_3616 | Tryptophan synthase beta chain (EC 4.2.1.20) | 8.84E-07 | -1.3 |
| U5S2V9 | CLAU_3655 | Aldehyde-alcohol dehydrogenase | 1.13E-12 | -2.03 |
| U5S2V9 | CLAU_3655 | Aldehyde-alcohol dehydrogenase | 4.17E-20 | -2.33 |
| U5S2J6 | CLAU_3721 | Rubrerythrin | 1.17E-04 | 1.28 |
| U5RZ45 | CLAU_3758 | Glutamate synthase (Ferredoxin) (EC 1.4.7.1) | 1.03E-02 | -1.09 |
| U5S336 | CLAU_3760 | Dipeptidase | 9.73E-11 | 1.37 |
| U5RZT6 | CLAU_3809 | Microcompartments protein | 3.19E-04 | 1.35 |
| U5RZF0 | CLAU_3821 | 4-hydroxy-tetrahydrodipicolinate reductase (HTPA reductase) (EC 1.17.1.8) | 1.63E-04 | 1.77 |
| U5RZV4 | CLAU_3828 | Cytidine deaminase (EC 3.5.4.5) | 1.42E-09 | -1.18 |
| U5S394 | CLAU_3829 | Deoxyribose-phosphate aldolase (DERA) (EC 4.1.2.4) (2-deoxy-D-ribose 5-phosphate aldolase) (Phosphodeoxyriboaldolase) (Deoxyriboaldolase) | 6.94E-08 | -1.21 |
| U5RZA4 | CLAU_3832 | Pyrimidine-nucleoside phosphorylase (EC 2.4.2.2) | 1.27E-56 | -1.3 |
| U5RZH4 | CLAU_3846 | Taurine-transporting ATPase (EC 3.6.3.36) | 2.72E-05 | 1.45 |
| U5RZB9 | CLAU_3847 | ABC-type transporter, periplasmic subunit family 3 | 3.32E-11 | 1.77 |
| U5RZX4 | CLAU_3848 | Uncharacterized protein | 7.35E-12 | 2.17 |
| U5RZJ2 | CLAU_3861 | Alcohol dehydrogenase (EC 1.1.1.1) | 7.72E-15 | 1.33 |

**References**

Bengelsdorf, F. R. *et al.* (2016) ‘Industrial acetogenic biocatalysts: A comparative metabolic and genomic analysis’, *Frontiers in Microbiology*, 7(JUL), pp. 1–15. doi: 10.3389/fmicb.2016.01036.

Huhnke, Raymond L.; Lewis, Randy S.; Tanner, R. S. (2008) ‘Isolation and Characterization of novel Clostridial Species (EP2061872A2)’, *Patent*.

Humphreys, C. M. *et al.* (2015) ‘Whole genome sequence and manual annotation of Clostridium autoethanogenum, an industrially relevant bacterium’, *BMC Genomics*, 16(1). doi: 10.1186/s12864-015-2287-5.

Jeong, Y. *et al.* (2014) ‘Draft Genome Sequence of Acid-Tolerant Clostridium drakei SL1 T , a Potential Chemical Producer through Syngas Fermentation’, *Genome Announcements*, 2(3), pp. 4–5. doi: 10.1128/genomeA.00387-14.

Köpke, M. *et al.* (2010) ‘Clostridium ljungdahlii represents a microbial production platform based on syngas.’, *Proceedings of the National Academy of Sciences of the United States of America*, 107(29), pp. 13087–92. doi: 10.1073/pnas.1004716107.

Mock, J. *et al.* (2015) ‘Energy conservation associated with ethanol formation from H2 and CO2 in Clostridium autoethanogenum involving electron bifurcation’, *Journal of Bacteriology*, 197(18), pp. 2965–2980. doi: 10.1128/JB.00399-15.

Ow, S. Y. *et al.* (2009) ‘iTRAQ underestimation in simple and complex mixtures: “the good, the bad and the ugly”.’, *Journal of proteome research*, 8(11), pp. 5347–55. doi: 10.1021/pr900634c.

Pham, T. K. *et al.* (2010) ‘A quantitative proteomic analysis of biofilm adaptation by the periodontal pathogen Tannerella forsythia’, *Proteomics*, 10(17), pp. 3130–3141. doi: 10.1002/pmic.200900448.

Raut, M. P. *et al.* (2016) ‘Quantitative proteomic analysis of the influence of lignin on biofuel production by Clostridium acetobutylicum ATCC 824.’, *Biotechnology for biofuels*, 9(1), p. 113. doi: 10.1186/s13068-016-0523-0.

Wang, S. *et al.* (2013) ‘NADP-Specific Electron-Bifurcating [FeFe]-Hydrogenase in a Functional Complex with Formate Dehydrogenase in Clostridium autoethanogenum Grown on CO’, *Journal of Bacteriology*, 195(19), pp. 4373–4386. doi: 10.1128/JB.00678-13.

Zhu, Z. *et al.* (2015) ‘Complete genome sequence of a malodorant-producing acetogen, Clostridium scatologenes ATCC 25775^T^’, *Journal of Biotechnology*, 212, pp. 19–20. doi: 10.1016/j.jbiotec.2015.07.013.
